# Spectral flow cytometry cluster analysis of therapeutic donor lymphocyte infusions identifies T cell subsets associated with outcome in patients with AML relapse

**DOI:** 10.3389/fimmu.2022.999163

**Published:** 2022-10-05

**Authors:** Ivan Odak, Ruth Sikora, Lennart Riemann, Lâle M. Bayir, Maleen Beck, Melanie Drenker, Yankai Xiao, Jessica Schneider, Elke Dammann, Michael Stadler, Matthias Eder, Arnold Ganser, Reinhold Förster, Christian Koenecke, Christian R. Schultze-Florey

**Affiliations:** ^1^ Institute of Immunology, Hannover Medical School, Hannover, Germany; ^2^ Department of Hematology, Hemostasis, Oncology and Stem Cell Transplantation, Hannover Medical School, Hannover, Germany; ^3^ Department of Pediatric Pneumology, Allergology and Neonatology, Hannover Medical School, Hannover, Germany

**Keywords:** donor lymphocyte infusion (DLI), immunotherapy, graft-versus-leukemia, allogeneic hematopoietic stem cell transplantation, acute myeloid leukemia, T cells, spectral flow cytometry, exhaustion markers

## Abstract

Identification of immune phenotypes linked to durable graft-versus-leukemia (GVL) response following donor lymphocyte infusions (DLI) is of high clinical relevance. In this prospective observational study of 13 AML relapse patients receiving therapeutic DLI, we longitudinally investigated changes in differentiation stages and exhaustion markers of T cell subsets using cluster analysis of 30-color spectral flow cytometry during 24 months follow-up. DLI cell products and patient samples after DLI were analyzed and correlated to the clinical outcome. Analysis of DLI cell products revealed heterogeneity in the proportions of naïve and antigen experienced T cells. Cell products containing lower levels of effector memory (eff/m) cells and higher amounts of naïve CD4^+^ and CD8^+^ T cells were associated with long-term remission. Furthermore, investigation of patient blood samples early after DLI showed that patients relapsing during the study period, had higher levels of CD4^+^ eff/m T cells and expressed a mosaic of surface molecules implying an exhausted functional state. Of note, this observation preceded the clinical diagnosis of relapse by five months. On the other hand, patients with continuous remission retained lower levels of exhausted CD4^+^ eff/m T cells more than four months post DLI. Moreover, lower frequencies of exhausted CD8^+^ eff/m T cells as well as higher amounts of CD4^+^temra CD45RO^+^ T cells were present in this group. These results imply the formation of functional long-term memory pool of T cells. Finally, unbiased sample analysis showed that DLI cell products with low levels of eff/m cells both in CD4^+^ and CD8^+^ T cell subpopulations associate with a lower relapse incidence. Additionally, competing risk analysis of patient samples taken early after DLI revealed that patients with high amounts of exhausted CD4^+^ eff/m T cells in their blood exhibited significantly higher rates of relapse. In conclusion, differentially activated T cell clusters, both in the DLI product and in patients post infusion, were associated with AML relapse after DLI. Our study suggests that differences in DLI cell product composition might influence GVL. In-depth monitoring of T cell dynamics post DLI might increase safety and efficacy of this immunotherapy, while further studies are needed to assess the functionality of T cells found in the DLI.

## Introduction

Donor lymphocyte infusions (DLI) are considered as curative treatment option for relapse of acute myeloid leukemia (AML) after allogeneic hematopoietic stem cell transplantation (alloHSCT) (1–3). The donor T cells are transferred to (re)-induce a graft-versus-leukemia (GVL) reaction eliminating the recurring malignant cells (4). Current practice of DLI treatment consists of leukapheresis to obtain donor lymphocytes which are then given in increasing dosages (5, 6) until remission is achieved or graft-versus-host disease (GVHD) is detected (7–9). While it has been shown that antineoplastic treatment (i.e. chemotherapy) alone can induce durable remission only in a very small amount of patients, the combination of antileukemic therapy and therapeutic DLI (tDLI) results in much higher response rates (1, 10, 11).

Many leukocyte subsets have been shown to contribute to GVL, however the main effect seems to be driven by CD8^+^ T cells (5, 12–14). Besides the numbers of CD3^+^, and in some centers CD4^+^ and CD8^+^ T cells, no further details on the composition of donor lymphocyte cell product are assessed in clinical practice.

The cell composition of DLI underlies a high heterogeneity (15, 16). Several approaches have been made to manipulate or select the content of the DLI in order to improve patients’ outcome. These vary from *in vivo* to *ex vivo* manipulation, such as unspecific T cell stimulation (17–23), enrichment of CD4^+^ T cells (24, 25), depletion of regulatory CD4^+^/CD25^+^ T cells (26, 27), and NK cell enriched/selected DLI (28, 29). There are also approaches to apply leukemia antigen-specific DLI (30, 31). Despite some promising results (6), identification of a key immune cell population either present in the DLI, or found at some time point post DLI failed. Therefore, standard of care remains an unmanipulated mixture of donor lymphocytes, solely scaled by the amount of T cells.

Studies investigating T cells and their ability to control the malignant cells added to the understanding of the underlying mechanism of GVL (25, 32–34). Deep phenotyping of surface molecules including exhaustion markers may provide a powerful tool to study differentiation stages to further dissect the role of the involved T cells.

In this study, we hypothesize that differences in T cell subsets of the DLI play a major role in eliciting a durable remission in AML patients. To test this, we use recently available 30-color spectral flow cytometry for in depth identification of differentiation stages and exhaustion markers of T cell subsets in DLI cell products itself and longitudinally in AML relapse patients receiving tDLI after matched alloHSCT. We analyzed the DLI cell products and patient samples after DLI during a 24 months follow up. We correlated the changes in T cell subsets with the occurrence of AML relapse post DLI. Finally, we assess the identified T cell clusters for their potential to predict relapse incidence using a competing risk analysis. In summary, the presented study is intended to identify phenotypical differences within the T cell compartment of DLI cell products as well as in patient samples post DLI associated with differential outcome to tDLI and thus to identify possible biomarkers.

## Materials and methods

### Cohort and study design

Our cohort consisted of 56 patients receiving unmanipulated DLI, applied between 2015 and 2019 in the department of Hematology, Hemostasis, Oncology and Stem Cell Transplantation at Hannover Medical School. The institutional review board approved the study (#2604-2015) and all study participants gave their written informed consent. In order to obtain a homogenous patient cohort, only relapsed *de novo* or secondary AML patients treated with therapeutic DLI of a fully HLA-matched donor were included in the study, resulting in a cohort of 13 patients. For all of them a sample of the DLI cell product was available. Details of the study recruitment, exclusion criteria and sampling are given in **Supplementary Figures 1**, **2**.

The indication for tDLI was a hematologic or molecular relapse after alloHSCT. Disease risk was assessed in accordance to ELN criteria (35). Post DLI, the recipients were regularly scheduled for outpatient visits, including clinical and laboratory-based (cytologic evaluation, MRD and high-sensitivity chimerism analyses) assessments of response to DLI. Dose-escalated (0.5-1 log increase of CD3^+^ cells/kg BW) subsequent DLI were applied if no complete remission was achieved after 4-6 weeks post DLI and no clinical signs of GVHD were detected. All patient samples post DLI were rated for presence of GVL *via* minimal residual disease chimerism analysis and as defined elsewhere (14) at the time point of sampling. In short, GVL was noted either when relative decrease by ≥50% or absolute decrease by ≥1.00% of host chimerism compared to the previous time point, conversion of MRD from positive to negative or decrease of MRD level by ≥10-fold occurred. Once GVL criteria have been met, stable host chimerism after GVL together with no relapse or absence of progressive disease at subsequent time points was also counted as GVL. In contrast, no GVL was defined as no changes or increase in host chimerism that fulfilled GVL criteria, and/or stable or ≥10-fold decrease of MRD, and/or relapse or progressive disease. Acute GVHD (aGVHD) and overlap GVHD and chronic GVHD (cGVHD) were diagnosed based on the EBMT-NIH-CIBMTR task force panel recommendation (36), applying the Mount Sinai Acute GVHD International Consortium (MAGIC) criteria for aGVHD (37) and the National Institutes of Health (NIH) 2014 criteria for cGVHD (38).

### Flow cytometry

Peripheral blood mononuclear cells (PBMCs) were prepared from patient whole blood samples using Ficoll gradient as described elsewhere (39) and stored in -80°C until further processing. After thawing, samples were stained for 30 min at room temperature with an antibody mix containing 29 monoclonal antibodies and a viability dye for cell phenotyping assessment of functional parameters including negative checkpoint regulators (NCRs) and costimulatory molecules (**Supplementary Table S1**). After washing, samples were acquired on an Aurora spectral flow cytometer (Cytek) equipped with five lasers (355 nm, 405 nm, 488 nm, 561 nm and 640 nm) using SpectroFlo version 2.2.0 (Cytek). All flow cytometry data were analyzed with FCS Express™ 7 (Denovo) and R (version 4.1, https://www.R-project.org/).

### Uniform manifold approximation and projection (UMAP) analysis

For UMAP analysis, conventional 2D gating was used to gate on T cells (**Supplementary Figure 3**). Due to low resolution capacity of the used CCR3 and CCR5 antibodies, these two markers were excluded from the further analysis. The cytometry data were then loaded into R and transformed using the logicle function. This was followed by data quality control/cleaning step using the PeacoQC algorithm (40). Clustering was then performed using the FlowSOM algorithm with default settings (41). The cluster analyses were separately done for the DLI cell product samples and all patient samples. The number of meta-clusters was set to 17 for the DLI cell product samples and to 20 for the patient samples. Dimensionality reduction was performed using the UMAP (**Figures 2A**, **3A**) approach. Cluster annotation was performed manually using the relative expression of all markers across clusters (**Supplementary Figures 4A, B**).

### Statistical analysis

Statistical analysis of data was performed with Prism 7 (GraphPad). Data was tested for normality distribution (Shapiro- Wilk test). For group comparisons of normally distributed data an unpaired two-tailed Student’s t-test, and for not normally distributed data a two-tailed, exact Mann-Whitney test was used. Longitudinal changes within the same patient were analyzed with paired Student’s t-test (normally distributed data) or two-tailed Wilcoxon matched-pairs signed ranked test (non-normally distributed data). Univariate analysis of clinical variables was performed with Student’s t-test or Mann-Whitney test for numerical variables (two-tailed, exact) and with Fisher’s exact test for categorical variables. Competing risk analysis of means of cumulative incidence curves of relapse (CIR) and non-relapse mortality (NRM) was performed with Gray’s test (42, 43). Grouping for competing risk analyses was based on high vs. low levels (frequencies and absolute counts) of identified T cell clusters. Separation of the groups respected the distribution of each cluster aiming at balanced group sizes while avoiding separation of adjacent values. P values <0.05 were considered significant.

## Results

### Cohort characteristics

**Figure 1:**
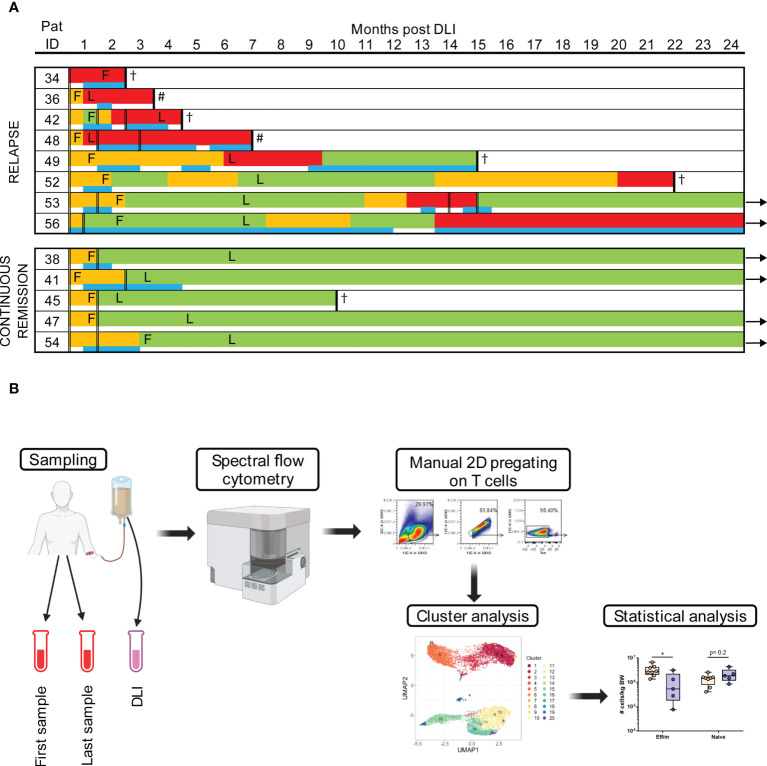
Study work flow and response to DLI. **(A)** Every patient is represented by one line with color coding giving details of timing and duration of GVL (green), noGVL (orange) and relapse (red) and the timing of concomitant antineoplastic treatment (blue). Application of DLIs is shown with double lines, timing of the first (F) and last (L) samples post DLI is marked in the time line of each patient. Follow-up time was 24 months. Arrows indicate continued survival beyond the study follow up, # indicates second allogeneic hematopoietic stem cell transplantation, † indicates death. **(B)** The workflow is shown. After sampling, PBMCs were measured with spectral flow cytometry. Data was then manually pre-gated on T cells for cluster analysis, which were subsequently statistically evaluated. Figure 1B was created with BioRender.com.

Of 13 participants meeting inclusion criteria, eight patients had a relapse of AML after a median of 4 months (1-20 months) post DLI, while five patients achieved a continuous remission during the two year follow-up (**Figure 1A**; **Table 1A**). Thus, the applied grouping of continuous remission and relapse was based on outcome post DLI. Additional information on patients’ disease and transplant characteristics are shown in **Supplementary Table S2** and further details about the DLI are given in **Table 1B**. Detailed information about the response to DLI for every individual patient is shown in **Supplementary Table S3**. Patients with continuous remission after DLI had a significantly longer overall survival (median of 24 months vs. 11 months), were older, received their DLI later after alloHSCT, but did not receive a higher dose nor total number of DLI (**Tables 1A, B**). The median donor age to patients with continuous remission was slightly lower, but did not reach statistical significance (**Table 1A**).

**Table 1A:** Cohort overview and response to DLI.

			All	Relapse		Continuous Remission					
N			13	8		5					
Age at DLI, y			57 (22–75)	51 (24–61)		67 (22-75)					
Sex, male			6 (46)	4 (50)		2 (40)					
Disease Details, sAML			2 (15)	1 (13)		1 (20)					
Disease risk, adverse risk*			4 (31)	3 (38)		1 (20)					
Donor age at alloHSCT, y			33 (24-58)	36 (28-43)		30 (24-58)					
Donor sex, male			9 (69)	5 (63)		4 (80)					
Conditioning regimen, RIC			6 (46)	3 (38)		3 (60)					
aGVHD post alloHSCT			4 (31)	3 (38)		1 (20)					
cGVHD post alloHSCT			6 (46)	3(38)		3 (60)					
DLI Trigger: relapse -Hematological relapse -Molecular relapse			13 (100) -11 (85) -2 (15)	8 (100) -7 (88) -1 (13)		5 (100) -4 (80) -1 (20)					
Antineoplastic treatment pre DLI			13 (100)	8 (100)		5 (100)					
Response to antineoplastic treatment pre DLI*											
-CRi, iHC -PR -SD			-4 (31) -7 (54) -2 (15)	-1 (20) -5 (63) -2 (40)		-3 (60) -2 (40) -0 (0)					
First DLI, mo post alloHSCT			13 (3-47)	12 (3-47)		24 (6-28)					
Dose first DLI, CD3+/kg BW			1×10^7^ (1,5×10^6^-1×10^7^)	1×10^7^ (1,6×10^6^-1×10^7^)		1×10^7^ (1,5×10^6^-1×10^7^)					
Total number of DLI			2 (1-3)	2 (1-3)		2 (2)					
Cumulative amount of applied donor cells (CD3^+^/kg BW)			1.6x10^7^ (1.6x10^6^-6.5x10^7^)	4.55x10^7^ (1.6x10^6^-6.5x10^7^)		1.3x10^7^ (1.5x10^7^-6x10^7^)					
Antineoplastic treatment post DLI			11 (85)	8 (100)		3 (60)					
First GVL, mo post DLI			2 (1-3)	2 (1-3)		2 (2-3)					
aGVHD post DLI			5 (38)	3 (38)		2 (40)					
cGVHD post DLI			7 (54)	4 (50)		3 (60)					
Severe cGVHD post DLI			3 (23)	2 (25)		1 (20)					
Overlap GVHD post DLI			5 (38)	3 (38)		2 (40)					
Relapse, mo post DLI			4 (1-20)	4 (1-20)		–					
OS at 24 mo post DLI, mo			22 (2-24)	11 (2-24)		24 (10-24)					
Patients alive at 24 mo post DLI			6 (46)	2 (25)		4 (80)					

Given is the median (range) for continuous variables and the absolute number (%) for categorical variables. aGVHD, acute graft-versus-host disease; alloHSCT, allogeneic hematopoietic stem cell transplantation; BW, bodyweight; cGVHD, chronic graft-versus-host disease; CRi, complete remission with incomplete hematological recovery; CTx, chemotherapy; DLI, donor lymphocyte infusion; GVL, graft-versus-leukemia effect; iHC, increased host chimerism; mo, months; OS, overall survival; PR, partial remission; SD, stable disease; RIC, reduced intensity conditioning; sAML, secondary acute myeloid leukemia; y, years. *according to ELN response criteria (35).

**Table 1B:** DLI characteristics.

Patient ID	DLI Trigger	Relapse triggering thLI (months post alloHSCT)		Anti-neoplastic treatment pre DLI	Response to antineoplastic treatment pre DLI*		Days post antineoplastic treatment at 1^st^ DLI	First DLI (months post alloHSCT)	Dose 1^st^ DLI (CD3+/kg BW)	Collection method of 1^st^ DLI	Total number DLI
34	Cytological R	3		Aza	SD		14	4	1,6×10^6^	Cryopreserved unstimulated leukapheresis	1
36	Cytological R	44		FLA-IDA	SD		29	45	1×10^7^	Fresh unstimulated leukapheresis	1
38	Cytological R	27		FLA-IDA	CRi, iHC		35	28	1×10^7^	Cryopreserved unstimulated leukapheresis	2
41	Molecular R	4		FLA-IDA	PR		29	6	1×10^7^	Fresh unstimulated leukapheresis	2
42	Cytological R	46		CLAEG	PR		35	47	1×10^7^	Fresh unstimulated leukapheresis	2
45	Cytological R	6		DnR+AraC	CRi, iHC		70	8	1,5×10^6^	Fresh unstimulated leukapheresis	2
47	Cytological R	23		FLA-IDA	CRi, iHC		24	24	1×10^7^	Cryopreserved G-CSF stimulated leukapheresis	2
48	Cytological R	12		MEC	CRi, iHC		42	13	5×10^6^	Fresh unstimulated leukapheresis	3
49	Cytological R	2		Aza + Ven	PR		8	3	1×10^7^	Fresh unstimulated leukapheresis	1
52	Cytological R	9		Aza	PR		8	11	1×10^7^	Fresh unstimulated leukapheresis	1
53	Cytological R	12		Aza	PR		8	13	1×10^7^	Cryopreserved G-CSF stimulated leukapheresis	2
54	Cytological R	24		Aza	PR		13	25	5×10^6^	Cryopreserved unstimulated leukapheresis	2
56	Molecular R	2		Aza + Sor	PR		44	4	6×10^6^	Cryopreserved unstimulated leukapheresis	2

alloHSCT, allogeneic hematopoietic stem cell transplantation; Aza, Azacitidine; BW, bodyweight; CLAEG, Cladribine + Cytarabine; CRi, complete remission with incomplete haematological recovery; Daunorubicin, CTx, chemotherapy; DLI, donor lymphocyte infusion; DnR/AraC, Daunorubicin + Cytarabine; FLA-IDA, Fludarabine + Cytarabine + Idarubicin; iHC, increased host chimerism; MEC, Mitoxantrone + Etoposide + Cytarabine; PR, partial remission; R, Relapse; RD, residual disease; Sor, Sorafenib; Ven, Venetoclax. *according to ELN response criteria (35).

### Heterogeneity of T cell subsets in DLI cell products

**Figure 2:**
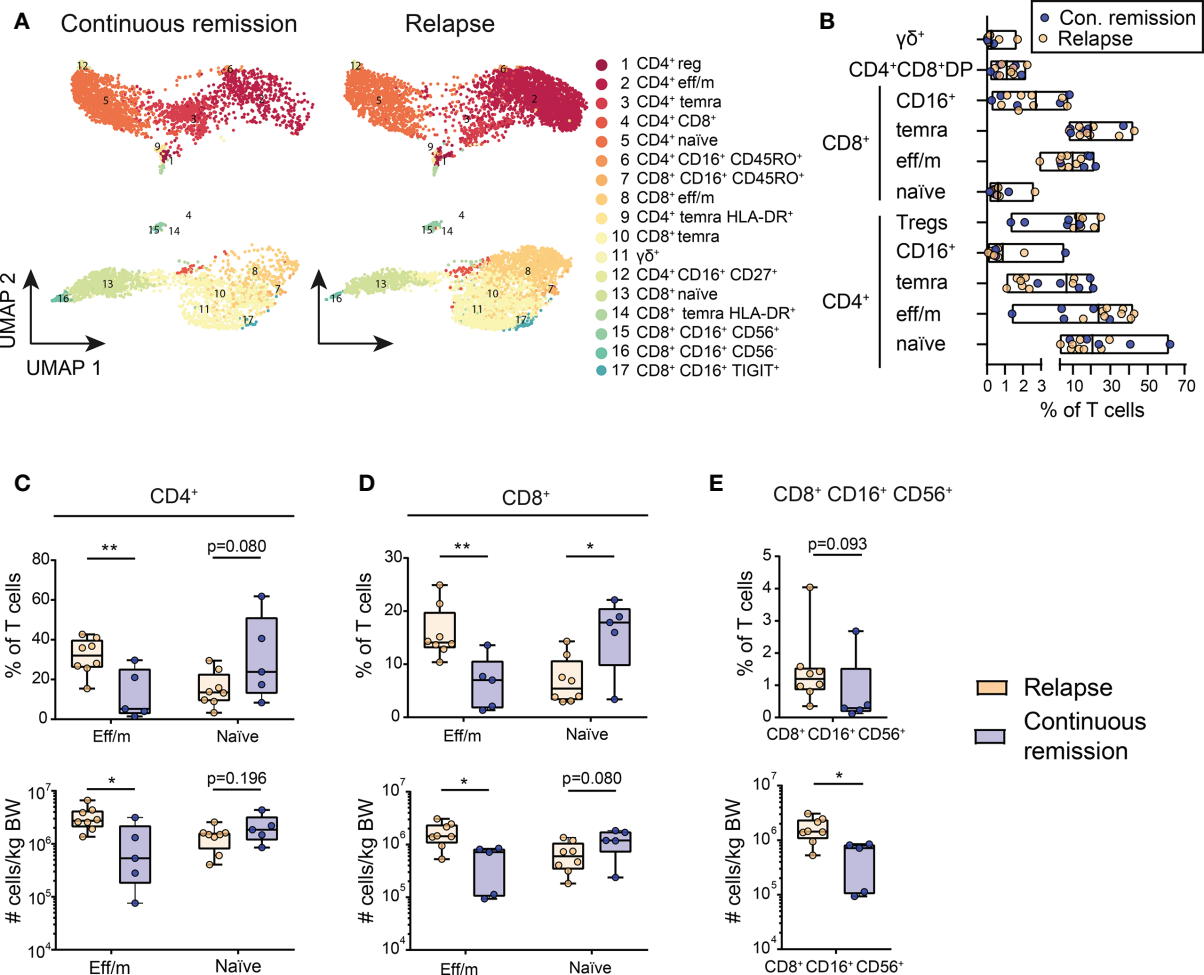
Differentially activated T cell clusters in the DLI cell product are associated with outcome. **(A)** Cluster analysis identified 17 different T cell populations within the DLI cell product. These are shown in Uniform Manifold Approximation and Projection (UMAP) for patients with continuous remission post DLI (n=5) and for patients with relapse post DLI (n=8) during the 24 months follow-up. The details on the 17 identified clusters are listed in **Supplementary Table S4**. **(B)** Composition of DLI cell products is shown, displaying the frequency of main T cell subsets. Each dot represents one DLI, with color-coding indicating the outcome to the cell product during a 24 months follow-up post DLI (blue = continuous remission, orange = relapse). Lines represent mean and bars represent the minimum and maximum. **(C–E)** Comparison of frequencies and cell counts of five clusters of the DLI cell product bags, with **(C)** showing results for CD4^+^ and **(D)** for CD8^+^ effector memory (eff/m) and naïve clusters and **(E)** CD8^+^ CD16^+^ CD56^+^ cluster. Grouping is based on the DLI cell product recipient’s outcome during a 24 months follow-up with continuous remission (blue, n=5) and relapse (orange, n=8). Clusters are named as in **(A)**. **(C–E)** Lines represent median, boxes represent 25th and 75th percentile and whiskers represent minimum and maximum. Statistical analysis **(C–E)** was performed by Student t test or Mann-Whitney test (two-tailed). *p<0.05, **p<0.001.

We performed an in-depth assessment of T cell subsets using spectral flow cytometry as depicted in the workflow (**Figure 1B**). An unsupervised UMAP analysis of the T cells revealed 17 distinct clusters in the DLI cell product (**Figure 2A**; **Supplementary Table S4**). Expression of all markers was manually evaluated across the whole cohort (**Supplementary Figure 4A**). For an overview of the broad composition of the cells found in the cell products, we sub grouped our T cell clusters based on their CD45RA and CCR7 expression (**Figure 2B**). Clusters 3 and 9 were classified as CD4^+^ temra, clusters 10 and 14 as CD8^+^ temra.

The most prevalent subsets were CD4^+^ effector memory (eff/m) T cells, with on average almost 25% of all T cells, followed by CD4^+^ naïve T cells (21%), while γδ^+^ T cells were the least frequent (mean 0.3%). The cell population with the highest variability amongst all cell products were the CD4^+^ naïve T cells (3.29-61.84%), followed by CD4^+^ eff/m T cells (1.40-42.67%). In total, 54% of all T cells were CD4^+^, while 45% were CD8^+^. Due to age-related changes on immune cell composition (44), usually younger donors are preferred (45). Therefore, we investigated the influence of the donor age on the T cell subsets in our cohort. Correlational analysis revealed that higher frequencies of CD8^+^ naïve cells (r=-0.69, p=0.012, **Supplementary Table S5**) associate with younger donor age. Taken together, analysis of the composition of the DLI cell products revealed a great heterogeneity of T cell subsets.

### Differentially activated T cell clusters in the DLI product are associated with outcome

In the next step, we tested whether the observed differences in the composition of the DLI can be associated with AML outcome. Expression of all markers was evaluated across the whole cohort stratified based on patient groups (**Supplementary Figures 5A, B**). Analysis of the proportion of T cell clusters in the DLI cell product revealed higher frequencies and numbers of CD4^+^ eff/m cells in patients with relapse after DLI. In the same group we found a trend towards decreased CD4^+^ naïve cells, however without reaching statistical significance (**Figure 2C**). Moreover, CD8^+^ eff/m cells were significantly higher in frequencies and numbers in the relapse group. At the same time, frequencies of CD8^+^ naïve cells in the same group were significantly reduced with a trend towards reduced absolute counts (**Figure 2D**). Of note, relapsing patients had received significantly higher counts of CD8^+^ CD16^+^ CD56^+^ T cells (**Figure 2E**). In sum, patients with continuous remission had received DLI cell products containing lower amounts of eff/m cells and higher amounts of naïve CD4^+^ and CD8^+^ T cells.

### Increased levels of exhausted effector memory T cells early after DLI are associated with relapse

**Figure 3:**
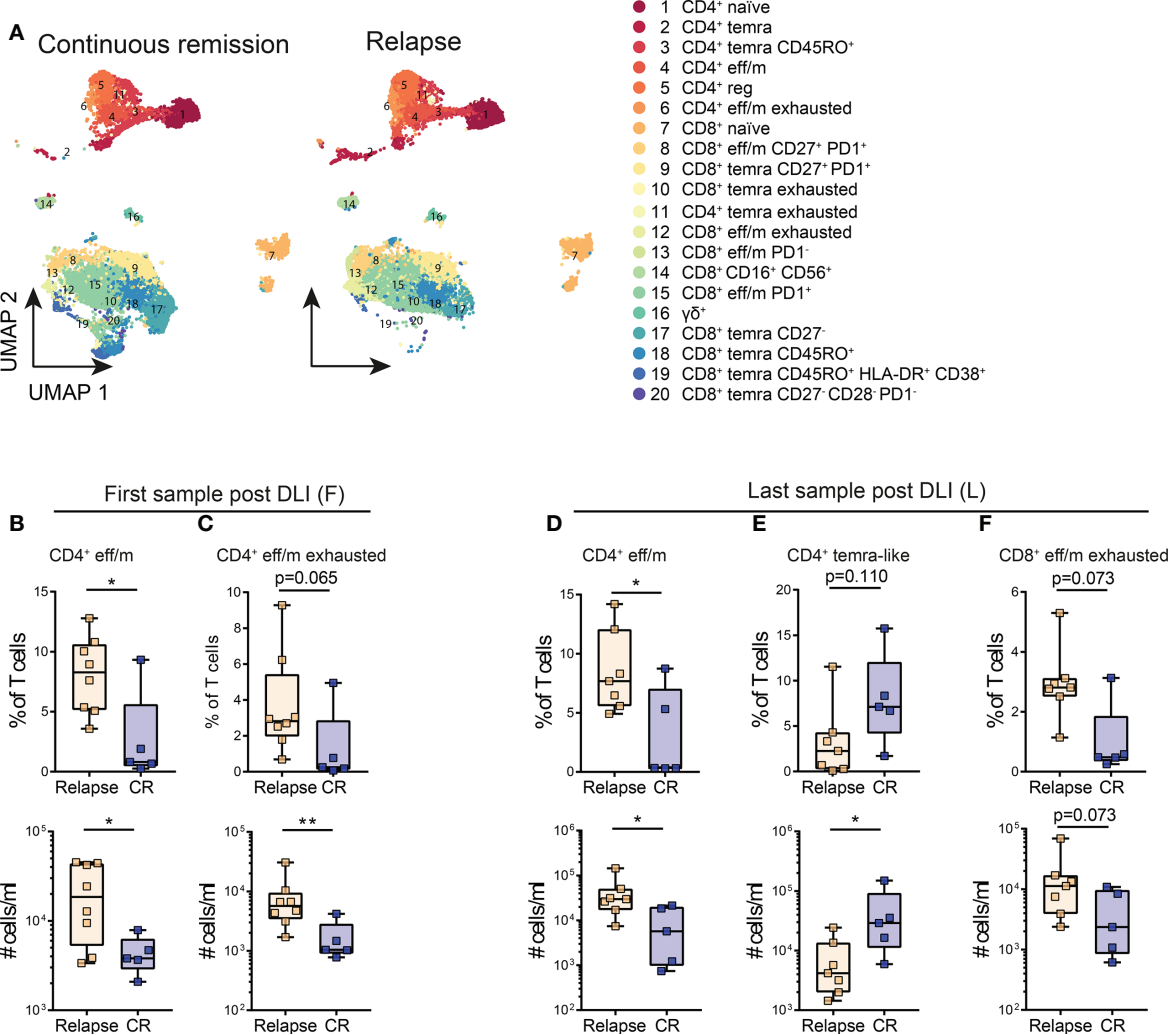
Lower levels of exhausted T cell clusters are associated with continuous remission early and late post DLI. **(A)** Cluster analysis identified 20 different T cell populations within the post-DLI samples. These are shown in Uniform Manifold Approximation and Projection (UMAP) including all samples of patients with continuous remission (n=5) and relapse (n=8) post DLI during the 24 months follow-up. The details on the 20 identified clusters are listed in **Supplementary Table S6**. **(B, C)** Comparison of frequencies and cell counts of two clusters identified in patient samples early after DLI between patients with continuous remission (blue, n=5) and relapse (orange, n=8) during the 24 months study follow-up. **(B)** Comparison of CD4^+^ eff/m and **(C)** exhausted CD4^+^ eff/m clusters between the groups. **(D–F)** Comparison of frequencies and cell counts of three clusters identified in patient samples late after DLI between patients with continuous remission (blue, n=5) and relapse (orange, n=7) during the 24 months study follow-up. **(D)** Comparison of CD4^+^ eff/m, **(E)** CD4^+^ temra CD45RO^+^ and **(F)** exhausted CD8^+^ eff/m clusters between the groups. All Clusters are named as in **(A)**. **(B–F)** Lines represent median, boxes represent 25th and 75th percentile and whiskers represent minimum and maximum. Statistical analysis **(B–F)**, was performed by Student t test or Mann-Whitney test (twotailed). *p<0.05, **p<0.001.

Next we analyzed T cell clusters at different time points following the DLI. Therefore, we performed an unsupervised cluster analysis with all patient samples post DLI, identifying 20 T cell clusters (**Figure 3A**; **Supplementary Table S6**). Expression of all markers was manually evaluated across the whole cohort (**Supplementary Figure 4B**) as well as stratified based on patient groups (**Supplementary Figures 6A, B**). First, we analyzed the T cell populations early after DLI (median 25 days, range 11-84 days) between patients with continuous remission compared to relapse (**Figure 1A**; **Supplementary Table S7**). At this time point, GVL effect was not yet present in patients without AML relapse, and was detected a median of 13 (range 4-51) days later (**Supplementary Table S7**). In the relapse group, only one patient was diagnosed with relapse at the same time point, while the other seven were diagnosed a median time of 150 (range 8-559) days later (**Figure 1A**; **Supplementary Table S7**). At this first time point post DLI, we identified a higher amount of CD4^+^ eff/m in patients with relapse post DLI (**Figure 3B**). Additionally, these patients had an exhausted (Tigit^+^ PD1^+^ HLA-DR^+^ CD38^+^ CD95^++^) CD4^+^ eff/m T cells phenotype in contrast to patients with continuous remission. These differences were detectable in both frequencies as well as absolute cell counts (**Figure 3C**). In summary, patients with relapse post DLI had higher levels of exhausted effector memory CD4^+^ T cells early after DLI, preceding the clinical diagnosis of relapse by a median time of 5 months.

### Lower levels of exhausted effector memory T cells associated with continuous remission persist a median of more than four months post DLI

Next, we investigated whether the T cell phenotype observed in samples taken at the last sampling time point is associated with response to DLI. Blood samples (n=12) were taken a median of 144 days (27-198 days) post DLI (**Supplementary Table S8**). At this time point, all patients with continuous remission had achieved GVL. In the relapse group, three patients were diagnosed with relapse at the time point of sampling, while the other five were diagnosed a median of 211 (186–396) days later (**Figure 1A**, **Supplementary Table S8**). Comparison of the last sample post DLI showed higher levels of CD4^+^ eff/m T cells in patients developing relapse post DLI (**Figure 3D**). Additionally, patients with continuous remission showed higher absolute counts of CD4^+^ temra CD45RO^+^ cells (CD45RO^+^ CCR7^-/lo^ CD27^++^ CD28^++^) compared to patients with relapse (**Figure 3E**). Moreover, within the relapse group we identified a trend towards increased cells with CD8^+^ eff/m exhausted phenotype (CD45RA^-^ CD45RO^+^ HLA-DR^+^ CD38^+^ Tigit^+^ PD1^++^, **Figure 3F**).

A longitudinal comparison (first vs. last sample post DLI) of the identified T cell populations separately for the groups showed that the T cell subpopulations retain a stable phenotype over time Moreover, no changes were detected in the CD4^+^ and CD8^+^ naïve T cell clusters and the T regulatory cells (**Supplementary Figure 7**). Taken together, a median of more than four months post DLI, we observed lower levels of likely functionally exhausted effector memory T cells in patients with continuous remission.

### Competing risk analysis of relapse incidence reveals predictive potential of identified T cell clusters

**Figure 4:**
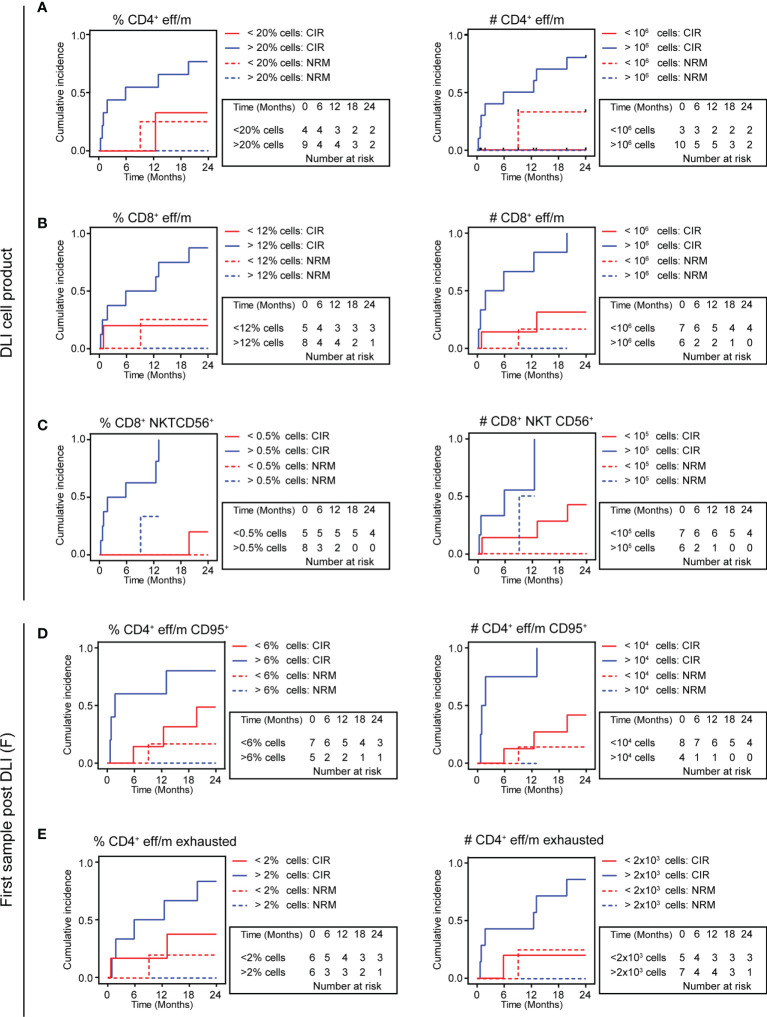
Relapse incidence is predicted by high vs. low eff/m T cell clusters. **(A–E)** Cumulative incidence curves (24 months follow-up) are displayed with relapse incidence (CIR, solid lines) and non-relapse mortality (NRM, dotted lines) as competing events for DLI cell products **(A–C)** or patient samples early after DLI **(D, E)**, discriminating between low (red) and high (blue) frequencies or absolute counts. **(A)** Shows in the left panel results for DLI cell products’ frequencies of CD4^+^ eff/m (>20% cells, n=9 vs. <20% cells, n=4, CIR, p=0.098; NRM, p=0.140), and in the right panel DLI cell products’ absolute counts of CD4^+^ eff/m (>10^6^ cells/ml, n=10 vs. <10^6^ cells/ml, n=3, CIR, p=0.039; NRM, p=0.071). **(B)** Shows in the left panel results for DLI cell products’ frequencies of CD8^+^ eff/m (>12% cells, n=6 vs. <12% cells, n=7, CIR, p=0.045; NRM, p=0.217), and in the right panel DLI cell products’ absolute counts of CD8^+^ eff/m (>10^6^ cells/ml, n=6 vs. <10^6^ cells/ml, n=7, CIR, p=0.009; NRM, p=0.382). **(C)** Shows in the left panel results for DLI cell products’ frequencies of CD8^+^ CD16^+^ CD56^+^ (>0.5% cells, n=8 vs. <0.5% cells, n=5, CIR, p=0.005; NRM, p=0.443), and in the right panel DLI cell products’ absolute counts of CD8^+^ CD16^+^ CD56^+^ (>10^5^ cells/ml, n=6 vs. <10^5^ cells/ml, n=7, CIR, p=0.023; NRM, p=0.308). **(D)** Shows in the left panel results for patient samples’ frequencies of CD4^+^ eff/m (>6% cells, n=5 vs. <6% cells, n=7, CIR, p=0.098; NRM, p=0.422), and in the right panel patient samples’ absolute counts of CD4^+^ eff/m (>10.000 cells/ml, n=4 vs. <10.000 cells/ml, n=8, CIR, p=0.004; NRM, p=0.536). **(E)** Shows in the left panel results for patient samples’ frequencies of exhausted CD4^+^ eff/m (>2% cells, n=6 vs. <2% cells, n=6 CIR, p=0.124; NRM, p=0.332), and in the right panel patient samples’ absolute counts of exhausted CD4^+^ eff/m (>2.000 cells/ml, n=7 vs. <2.000 cells/ml, n=5, CIR, p=0.046; NRM, p=0.248) and in the right panel. CIR and NRM were investigated by means of cumulative incidence curves employing Gray’s test.

To test whether the identified T cell clusters might be suited for prediction of AML outcome in the setting of DLI treatment, we categorized DLI cell product samples (n=13) based on their amount of T cells and regardless of the clinical outcome. Cumulative incidence of relapse (CIR) and non-relapse mortality (NRM) during the study period of 24 months were taken into consideration. Competing risk analysis revealed that in DLI cell products with higher levels (>10^6^ cells/kg BW, n=10) of the CD4^+^ eff/m cluster, the relapse incidence was significantly higher compared to low CD4^+^ eff/m containing DLI samples (p=0.039, **Figure 4A**) with a trend towards increased NRM (p=0.071). However, analyzing the CD4^+^ eff/m frequencies, we observed only a trend for increased relapse incidence in the high population (>20%, n=9) containing samples (p=0.098, **Figure 4A**). For the CD8^+^ eff/m cluster, higher levels (>10^6^ cells/kg BW, n=6) were associated with higher relapse incidence (p=0.009, **Figure 4B**). This result of increased relapse incidence was also seen for high (>12%, n=6) CD8^+^ eff/m frequencies (p=0.045, **Figure 4B**). No differences in the competing risk analysis of relapse incidence was seen in high vs. low CD4^+^ and CD8^+^ naïve cell population. Contrarily, the CD8^+^ CD16^+^ CD56^+^cluster revealed predictive potential, as cell counts >10^5^ cells/kg (n=6) or frequency >0.5% (n=8) were both significantly associated with higher relapse incidence (absolute counts p=0.023, frequency p=0.005, **Figure 4C**). Taken together, competing risk analysis of relapse incidence and NRM during a 24 months study period revealed the predictive potential of T cell subsets of the DLI cell product for relapse incidence.

Next, we investigated the predictive potential of patient samples early after DLI treatment. To do so, we categorized all first patient samples post DLI based on their amount of T cells for the previously identified T cell clusters. One patient was diagnosed with relapse at this time point and was thus excluded from the analysis, resulting in twelve samples eligible for analysis with a median sampling time point of 23 days post DLI (range 11-84) and median time until relapse of 150 days (range 14-559). CIR and NRM during the study period of 24 months were taken into consideration. Competing risk analysis revealed that in first patient samples post DLI with high CD4^+^ eff/m cell count (>10.000 cells/ml, n=4) the relapse incidence was significantly higher compared to low CD4^+^ eff/m containing patient samples (p=0.004, **Figure 4D**). This result of increased relapse incidence was also seen as a trend for high (>6%, n=5) CD4^+^ eff/m frequencies (p=0.098, **Figure 4D**). Moreover, we detected a significant higher relapse incidence in high (>2.000 cells/ml, n=7) CD4^+^ eff/m exhausted cluster containing patient samples (p=0.046, **Figure 4E**), but not for frequencies (>2%, p=0.124, **Figure 4E**). In sum, competing risk analysis of differentially expressed T cell clusters of patient samples predicted relapse at a median time of 5 months prior to actual diagnosis.

## Discussion

DLI treatment aims at (re)-induction of GVL by application of donor lymphocytes, commonly dosed by the amount of donor CD3^+^ cells per patient’s bodyweight without further characterization of lymphocyte subpopulations. While a dose-dependent effect of DLI on GVL (and GVHD) induction has been described (8), in our cohort there was no difference between relapsing patients and patients with continuous remission with regard to the dose of the first DLI, the total number of DLI and the cumulative amount of applied donor T cells (**Table 1A**). Thus, by employing a 30-marker spectral flow cytometry panel, we expand on the current DLI concept, showing in a cohort of 13 patients that differences within the T cell clusters of the DLI cell product as well as in patient samples post DLI are associated with response to the cell therapy.

An in-depth phenotyping of the DLI cell products and patients’ blood after DLI might identify cell populations as key for GVL activity. We and others recently demonstrated deep immune cell phenotyping using spectral flow cytometry in patient samples (46, 47). However, to fully utilize the power of this method, complementing the standard 2D gating approaches by more complex multi-modal analysis principles, such as uniform manifold approximation and projection (UMAP) allows for more complete dissection of the immune system (48, 49).

The UMAP analysis employed in our study identified 17 T cell subpopulations in the DLI cell products and 20 T cell subpopulations in the patient samples with distinct phenotypes. It is expected that number of subpopulations differ in DLI products as compared to patient material for several reasons, e.g. T cell space, expansion of immune cells, inflammation, infection, mixed chimerism when relapse is present, as well as interpatient differences, all leading to a higher complexity of the patient samples. Moreover, it cannot be ruled out that pre-analytic procedures such as cryopreservation might have impacted the expression of surface antigens. In the literature no influence of cryopreservation on the absolute T cell count is described (50). Nevertheless, a decrease in the Treg cell frequencies as a consequence of cryopreservation has been described (51), as well as marginal reduction of HLA-DR^+^ CD38^+^ and of CD45RA^+^ CD62L^+^ lymphocytes within CD4^+^ and CD8^+^ subsets (50). However, all biomaterial used in our study was cryopreserved following the same protocol and thus potential changes on expression of surface antigens should have occurred to all samples in equal measure.

The DLI cell products in our cohort exhibited a high level of T cell heterogeneity. This finding goes in line with a recent study by Ortí et al. that provided evidence of T cell variety of DLI cell products in that cohort (15). However, in contrast to our study, the authors could not find an association between T cell memory subsets in the DLI cell product and response to DLI. This might be due to differences in the panels used, as well as the fact that we only included AML patients. Upon in-depth characterization of the DLI cell products, we identified low amounts of eff/m cells and high amounts naïve cells both in CD4^+^ and CD8^+^ populations in relapse-free AML patients. This finding might support the hypothesis that long-term persistence of adaptive immunity leads to destruction of remaining AML blasts. Successful establishment of T cell memory relies on naïve cells by recognizing their cognate (leukemia) antigens. Therefore, a large number of naïve T cells capable of being successfully primed on malignant antigens would increase the likelihood of GVL induction (52). Nevertheless, depletion of naïve T cells in peripheral blood stem cell grafts in a trial aiming at reduction of GVHD did not lead to excessive rates of relapse (53). However, the different setting and underlying immunology needs to be taken into consideration as the overall population in that study was transplanted in complete remission while for tDLI relapse is a prerequisite. This is supported by a phase I study in which prophylactic DLI was administered to patients after depletion of naïve T cells. This intervention led to low incidence of GVHD, but to a rather low progression-free survival of only 50% (54). In sum, these findings suggest lower priming capacity in DLI cell products of relapsing patients.

Recently it has been reported that invariant NKT (iNKT) cells enriched DLI are potent killers of malignant cells *ex vivo* (55). However, since we did not include markers in our staining panel to be able to identify iNKT cells, the detected signal for higher levels of CD16^+^ CD56^+^ cells within the DLI cell products associated with relapse warrants further testing.

Within our prospective cohort study, we detected a T cell phenotype associated with long-term remission of AML after DLI. This phenotype showed similar characteristics to the one seen in remission inducing DLI cell products, with lower levels of eff/m T cells. Upon in-depth characterization of the T cell subsets found in patient samples early and late post DLI we identified T cell clusters with a mosaic of surface molecules implying an exhausted functional stage both in CD4^+^ and CD8^+^ T cells as associated with relapse. This is in line with the literature describing T cell exhaustion as a main factor for resistance to GVL (25, 34). Early after DLI (median of 25 days) we saw the exhausted T cell phenotype exclusively in the CD4^+^ compartment. This finding points towards the importance of CD4^+^ cells in supporting GVL induction (12, 24, 25, 56, 57).

The signature of low exhausted CD4^+^ eff/m cells in patients with durable AML remission showed long term persistence (median of 144 days). At this time point we also observed an association of increased numbers of CD4^+^ temra CD45RO^+^ cells with long lasting response to DLI compared to relapsing patients. Underlying mechanisms behind this observation might be multiple and not mutually exclusive. A pool likely containing AML-specific T cells in the group with continuous remission post DLI might be intrinsically more resistant to attainment of an anergic/exhausted phenotype. Alternatively, the CD4^+^ temra CD45RO^+^ T cells in the same group might have originated from the pool of naïve T cells present in the DLI cell product, encountered their cognate antigen, and proceeded to create bona fide long-lasting memory cells. In the relapsing patients, the same process might have occurred at some point, as indicated by intermittent GVL, but was lost due to T cell exhaustion. However, such hypotheses require further research in order to be validated, such as characterization of the exhaustion profile as well as *in vitro* cytotoxicity assays of T cells from responders vs. non-responders. This might pave the way towards identification of underlying mechanisms of the observed differences in T cell phenotypes regarding GVL induction. Investigation of these bona fide AML-specific T cells might pave the way to creation of long-lived T cells resistant to exhaustion signaling. Nonetheless, higher levels of CD8^+^ eff/m with an exhausted (CD45RA^-^ CD45RO^+^ HLA-DR^+^ CD38^+^ Tigit^+^ PD1^++^) phenotype in patients with AML relapse present only in the last time point post DLI, are pointing towards the importance of functional CD8^+^ eff/m for a long-lasting effective DLI response. This concurs with recent reports, showing a reversal of T cell exhaustion upon successful DLI treatment (25, 32, 33). However, longitudinal comparison of the T cell clusters between the first and last sampling post DLI did not yield any significant differences. This might imply the persistence of cell phenotypes in patients responding to DLI. On the other hand, it is also feasible that the magnitude of change of leukemia-specific T cells was too small to be observed due to our small cohort. While DLI cell products of patients with continuous remission had contained a higher proportion of naïve T cells compared to relapsing patients, we did not observe a difference between the groups in patient samples post DLI. This is in line with the literature, as naïve donor cells differentiate quickly upon antigen exposure (5).

Importantly, the T cell phenotypes associated with relapse during the 24 months follow-up were seen already at a median time of 5 months prior to the diagnosis of relapse, suggesting a potential as biomarker. This is further supported by the competing risk analysis of relapse incidence and NRM using an unbiased sample analysis approach comparing high vs. low frequency/count T cell cluster (**Figure 4**). However, given the heterogeneity of clinical courses including additional antineoplastic treatment concomitant to DLI therapy, we cannot rule out an impact on the observed T cell cluster results. Moreover, although not different between the groups (**Table 1A**), classical risk factors such as genetic risk and response to antineoplastic treatment (i.e. tumor burden) prior to DLI might play an important role with regard to response to DLI and might in some cases not be counteracted by DLI therapy. Additionally, HLA downregulation has been described as mechanism in AML immune evasion (58, 59). This has been further specified by identification of epigenetic modulation as main driver of the evasion mechanism, which holds the potential to be pharmacologically targeted (60). Considering the implication of GVL enhancement, future research should investigate HLA downregulation including epigenetic modulation as mechanism for failure of DLI therapy.

Our results are in line with the literature describing the importance of the functional state of immune cells for tumor control (25, 32–34, 59). Thus, reversal of immune cell exhaustion by immune-modulating drugs, such as checkpoint inhibitors might become a key intervention for enhancing treatment success, as there is emerging data supporting the role of check point inhibition for treatment of relapse after alloHSCT (61), also in combination with DLI (62).

Of note, albeit limited by the small sample size, the overall survival at 24 months post DLI in our cohort was with 46% in the upper range of published survival rates of cohorts with relapsed myeloid malignancies treated with antineoplastic treatment and tDLI, with reported two-year survival of 15-56% (1, 10, 63). Moreover, in line with a large DLI study which reported no association between cGVHD and reduce relapse incidence (64), we did not observe a difference in cGVHD incidence nor in cGVHD severity between patients with continuous remission vs relapse after DLI (**Table 1A**).

Our study results are limited by both small cohort and relatively short time of observation of 24 months. Given the limited statistical power, the results of our study are predominantly descriptive. Therefore, the identified T cell phenotypes require validation in a large validation cohort in order to replicate the findings and to statistically examine the results in a multivariate model, paving the way towards usage as biomarker. Additionally, in this study, we exclusively focused on T cells. Thus, underlying changes in non T cells, e.g. B cell subsets, cannot be ruled out and future studies should therefore aim not only to reproduce but also to investigate the whole lymphocyte subset repertoire with regard to DLI response. This future research might form the basis to develop an AML-specific immune cell enriched, highly effective DLI with an improved safety profile.

In conclusion, differentially activated T cell clusters, both in the DLI product and in patients after DLI treatment, are associated with response of tDLI treated AML relapse. Our study points towards the importance of the composition of the DLI cell product composition regarding GVL activity. In-depth monitoring of T cell dynamics post DLI might increase safety and efficacy of this immunotherapy.

## Data availability statement

The original contributions presented in the study are included in the article/**Supplementary Material**. Further inquiries can be directed to the corresponding authors.

## Ethics statement

The studies involving human participants were reviewed and approved by Ethikommission, Hannover Medical School. The patients/participants provided their written informed consent to participate in this study.

## Author contributions

IO, CRS-F, and CK planned and designed the study. MD and CRS-F collected and prepared the blood samples. CRS-F, RS, MB, JS, ED, MS, ME, and CK obtained the clinical patient data. RS, LB, and IO performed experiments. IO, RS, LR, and CRS-F analyzed the data. CRS-F, IO, YX, AG, RF, and CK discussed and interpreted the data. RS and IO drafted the figures. CRS-F and IO wrote the manuscript. All authors reviewed and edited the manuscript. All authors approved the submitted version.

## Funding

This work was supported by Deutsche Forschungsge-meinschaft (DFG, German research Foundation) Excellence Strategy EXC 2155 “RESIST” to RF (Project ID39087428), Deutsche Forschungsgemeinschaft SFB 900, project ID 158989968 (projects B1 to RF and B8 to CK) and the German Federal Ministry of Education and Research (01EO1302) to CRS-F and CK. LR was supported by the TITUS clinician-scientist program at Hannover Medical School, which is funded by the Else Kröner-Fresenius foundation.

## Conflict of interest

The authors declare that the research was conducted in the absence of any commercial or financial relationships that could be construed as a potential conflict of interest.

## Publisher’s note

All claims expressed in this article are solely those of the authors and do not necessarily represent those of their affiliated organizations, or those of the publisher, the editors and the reviewers. Any product that may be evaluated in this article, or claim that may be made by its manufacturer, is not guaranteed or endorsed by the publisher.
