# Supplementary material for: Spectral flow cytometry cluster analysis of therapeutic donor lymphocyte infusions identifies T cell subsets associated with outcome in patients with AML relapse
Source: Front Immunol. 2022 Oct 5;13:999163. doi: 10.3389/fimmu.2022.999163 (PMC9579313; doi:10.3389/fimmu.2022.999163)
Supplement: Supplementary file 1 [file DataSheet_1.docx]

**Supplementary Data**

Supplementary Figure 1. Details of study recruitment and number of samples.

Supplementary Figure 2. Sampling details.

Supplementary Figure 3. Gating strategy of spectral flow cytometry analysis.

Supplementary Figure 4. Relative antigen expression for each marker used in the cluster analysis.

Supplementary Figure 5. Ridgeplots of DLI cell products stratified by response to DLI.

Supplementary Figure 6. Ridgeplots of patient samples stratified by response to DLI.

Supplementary Figure 7. Longitudinal comparison of T cell clusters comparing first vs. last sampling time points.

Supplementary Table S1. Antibody panel for spectral flow cytometry.

Supplementary Table S2. Disease and transplant characteristics.

Supplementary Table S3. Details about response to DLI.

Supplementary Table S4. Cluster phenotypes of DLI cell products.

Supplementary Table S5: Correlation of DLI composition with donor characteristics.

Supplementary Table S6. Cluster phenotypes of patients’ samples.

Supplementary Table S7: Clinical characteristics of first samples post DLI.

Supplementary Table S8: Clinical characteristics of last samples post DLI.

**
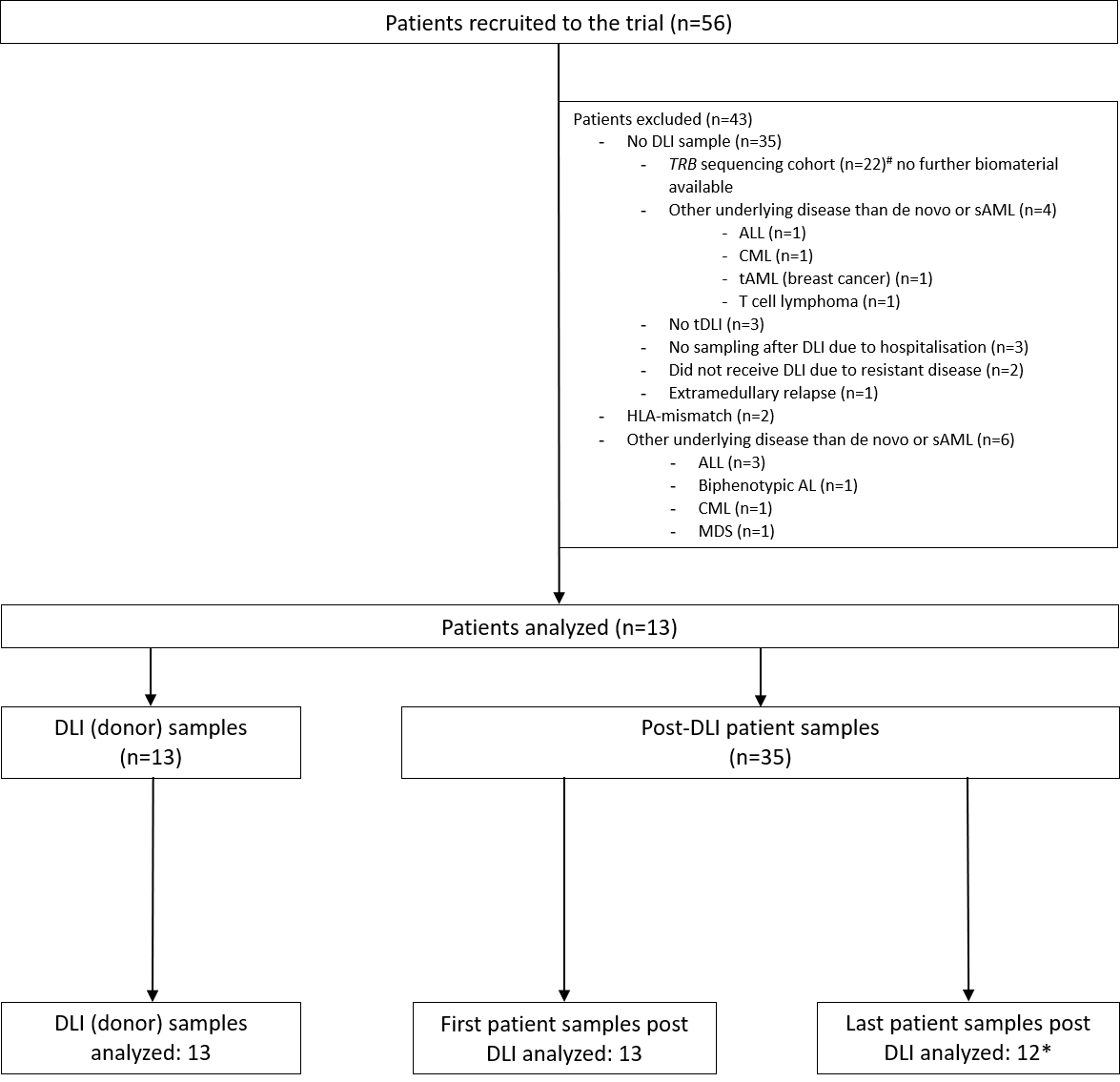
**

**Supplementary Figure 1. Study recruitment and number of samples.**

Abbreviations: AL acute leukemia, ALL acute lymphocytic leukemia, AML acute myeloid leukemia, CML chronic myeloid leukemia, DLI donor lymphocyte infusion, HLA human leukocyte antigen, sAML secondary AML, tAML therapy-related AML, tDLI therapeutic donor lymphocyte infusion, *TRB* t cell receptor beta.*Patient ID 34 had only one sample post DLI available. ^#^Corresponding publication (1) is listed under “References” at the end of the Supplementary Material.

**DLI**

**1**

**DLI**

**2**

**DLI**

**3**

**
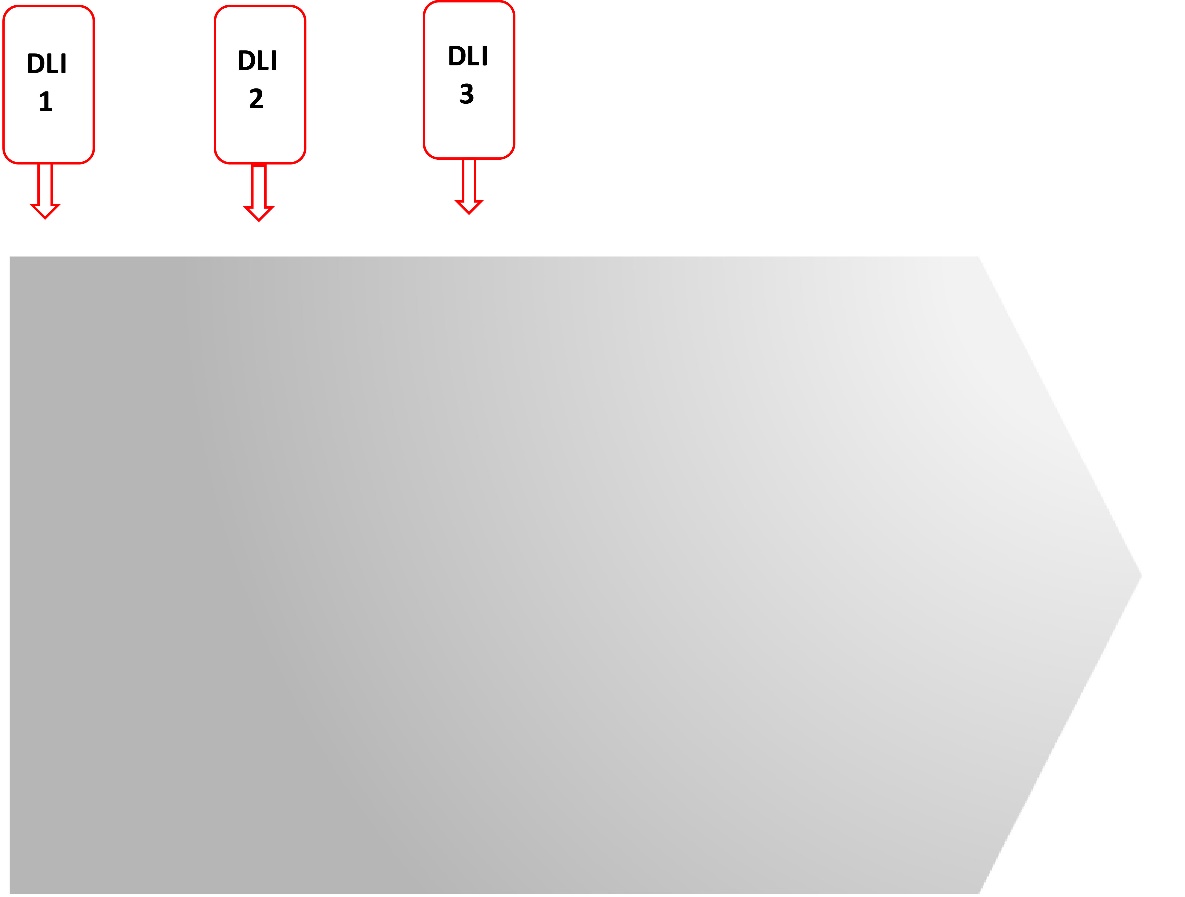
**

| Sampling time point: | d+14 | d+30 | d+60 | d+90 | d+120 | d+180 |
| --- | --- | --- | --- | --- | --- | --- |
| Sample name: | **A** | **B** | **C** | **D** | **E** | **F** |
| Number of samples: | 6 | 8 | 6 | 6 | 2 | 6 |
| Median day post-DLI (range): | 12  (11-21) | 28  (21-31) | 55  (47-62) | 87  (83-93) | 117  (114-120) | 179  (167-198) |
| Median days post-DLI at 1st occurrence of GVL if applicable (range): | 28  (-)  n=1 | 38  (31-44)  n=4 | 69  (62-88)  n=3 | - | - | - |
| Median difference between GVL detection and sampling in days if applicable (range): | - | - | 17  (0-27) | 49  (20-53) | 27  (-) | - |

**Supplementary Figure 2. Sampling details.**

Abbreviations: d days, DLI donor lymphocyte infusion, GVL graft-versus-leukemia.

**
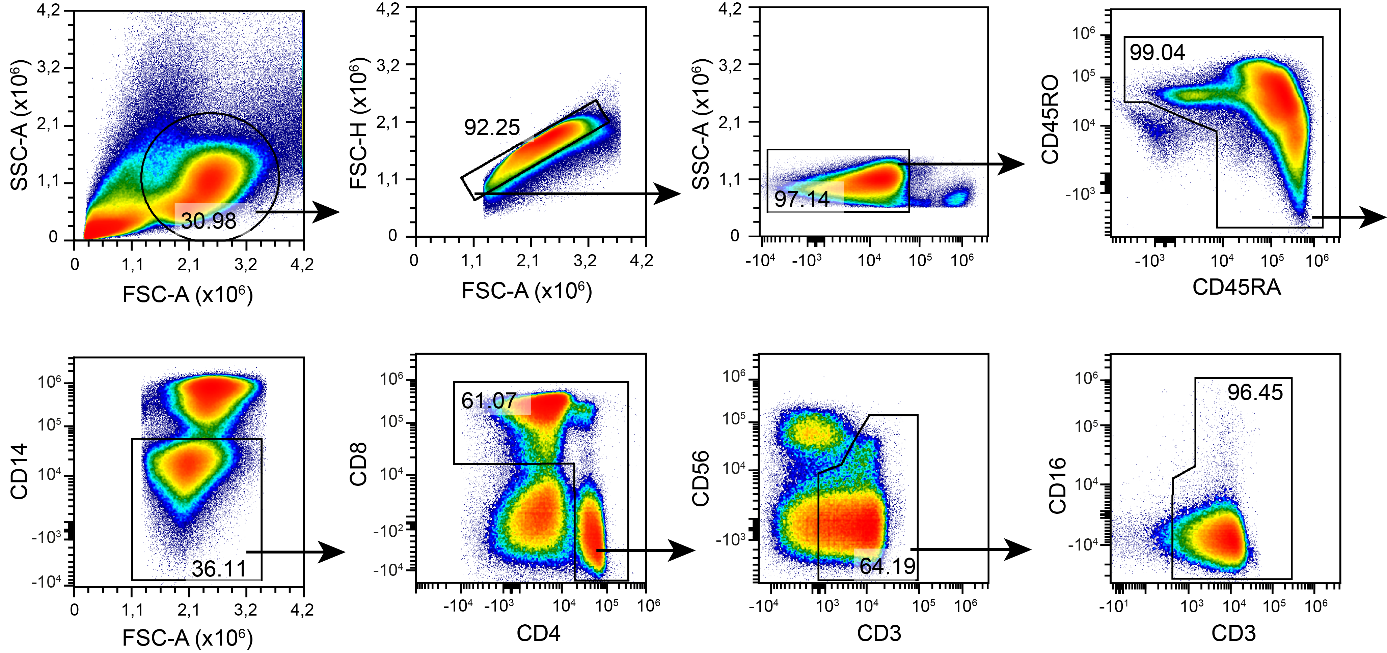
**

**Supplementary Figure 3. Gating strategy of spectral flow cytometry analysis.**

Shown is the spectral conventional 2D gating approach of flow cytometry data to gate on T cells for subsequent UMAP analysis.

**
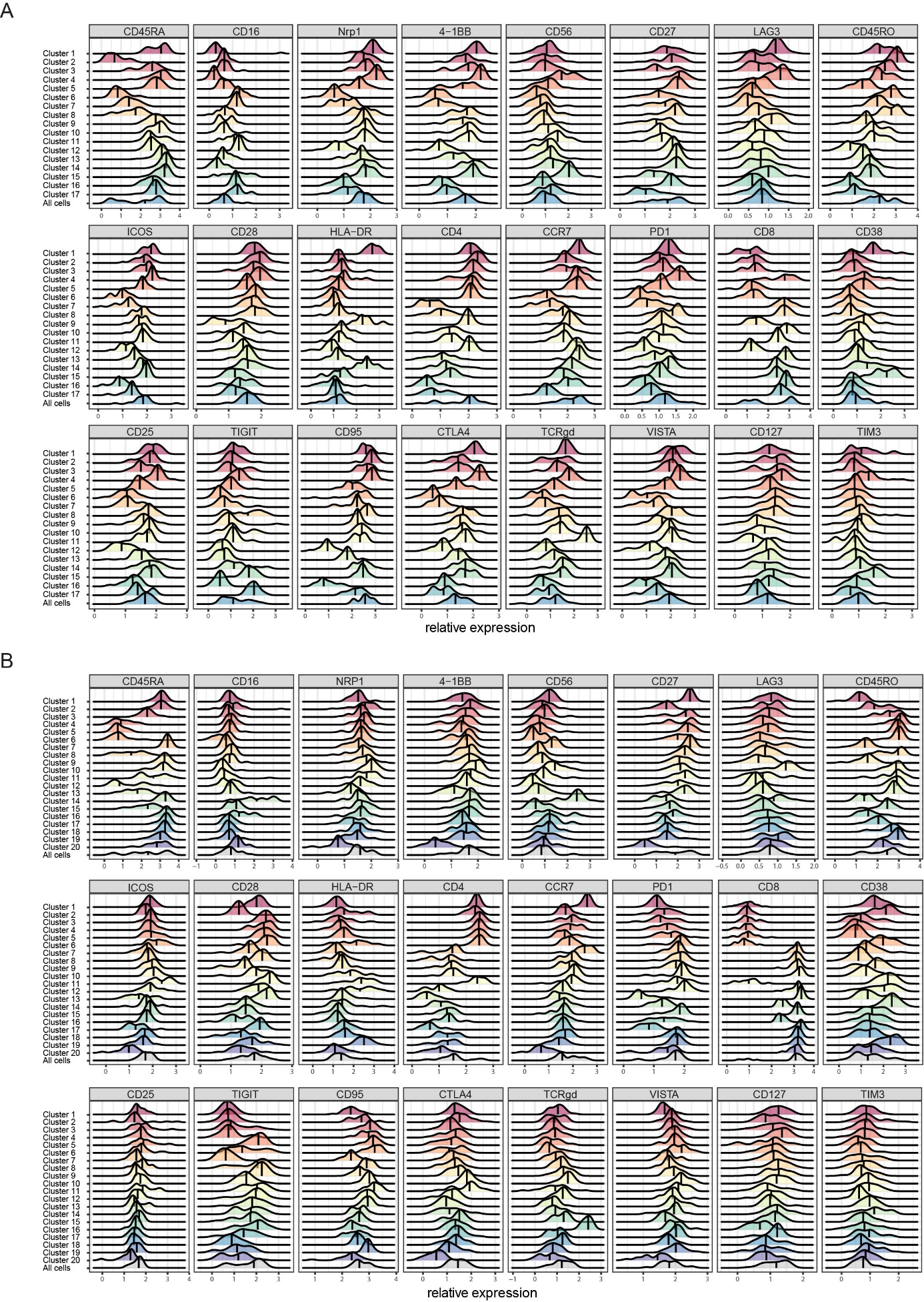
Supplementary Figure 4. Relative antigen expression for each marker used in the cluster analysis.**

Shown are single plots for each marker used in the cluster analysis, displaying the relative antigen expression (x axis) of the DLI cell products (A) and of the patient samples (B) for each of the clusters and for all the cells together.

**
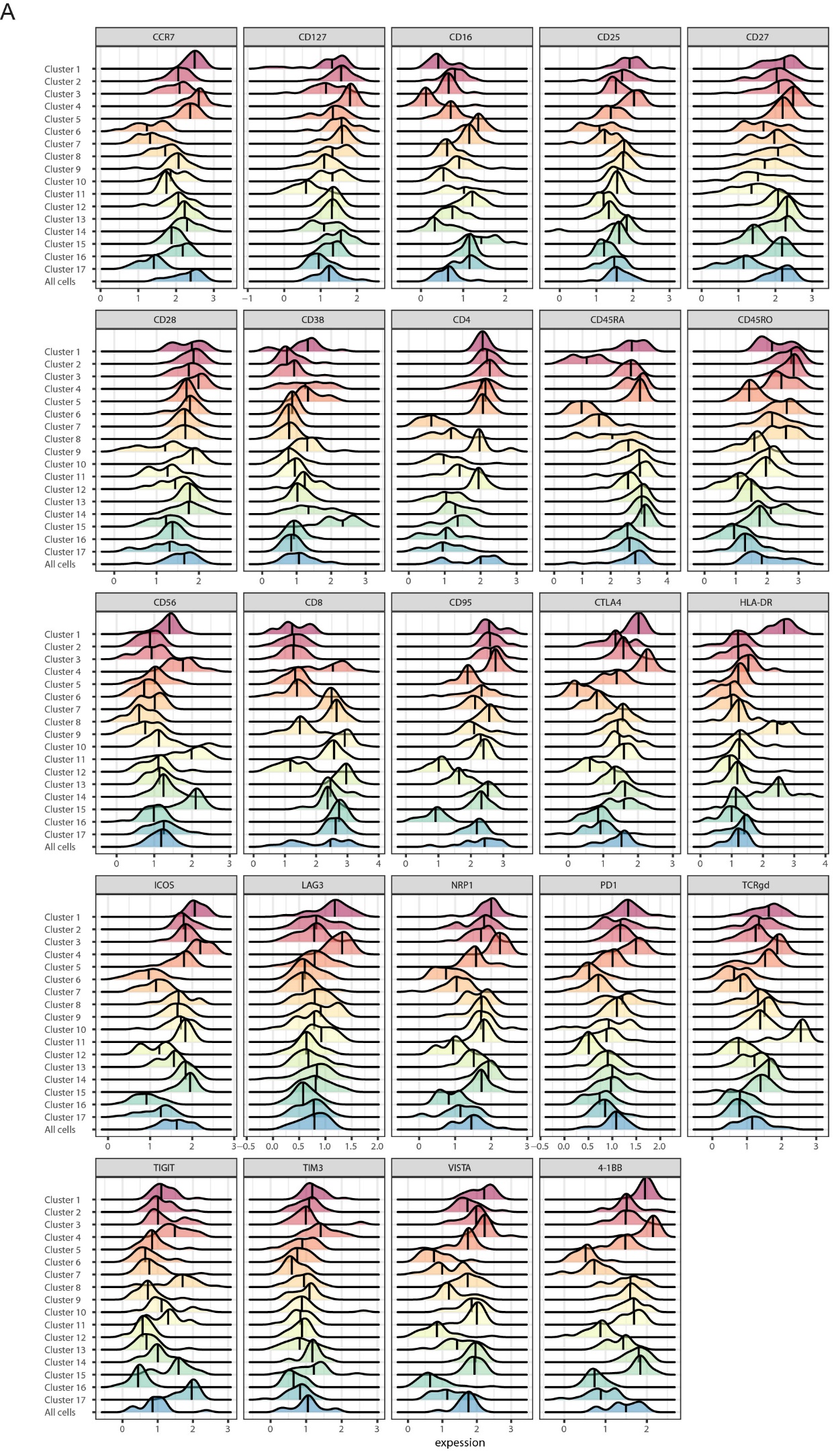
**

**
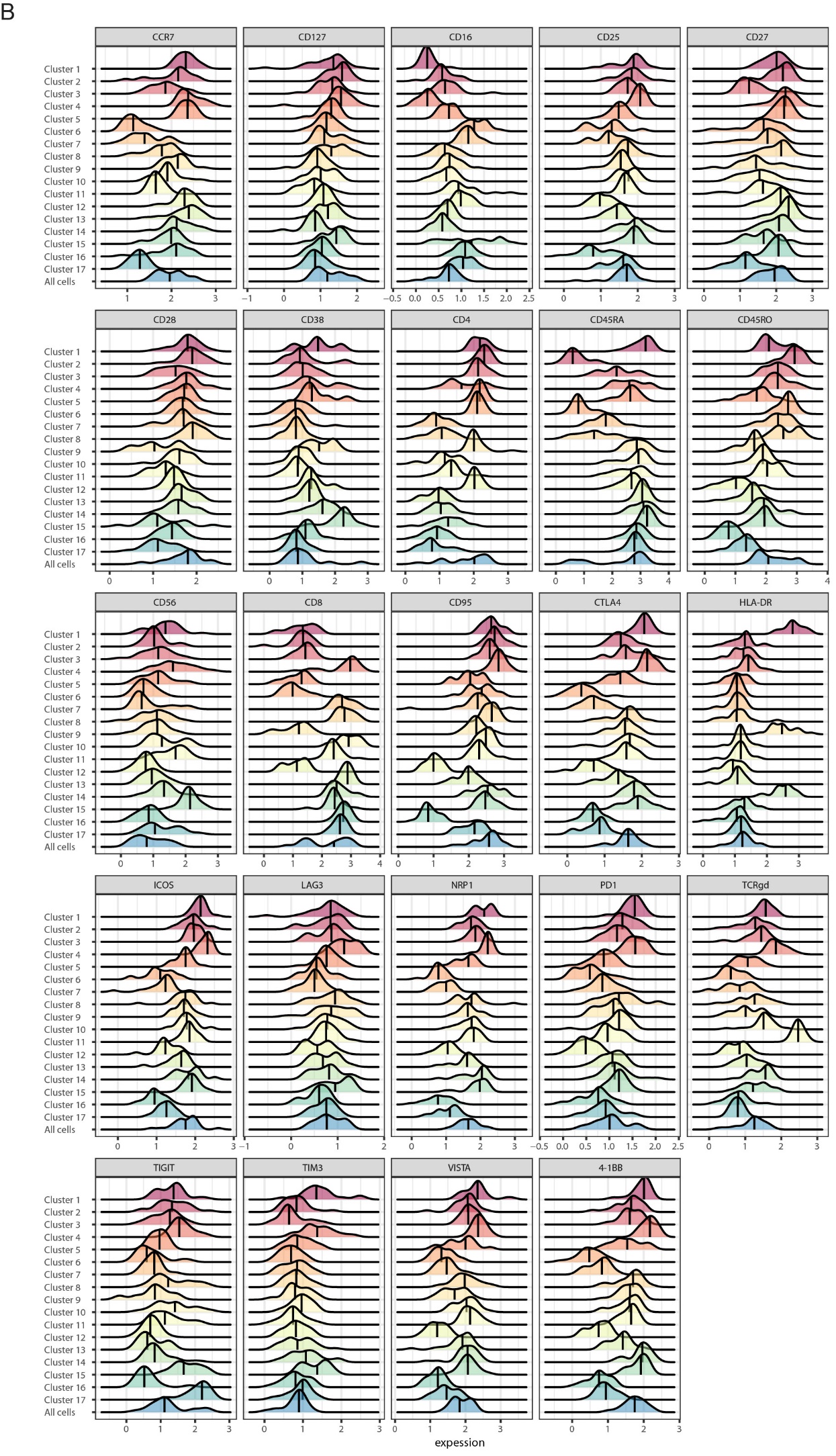
Supplementary Figure 5. Ridgeplots of DLI cell products stratified by response to DLI.**

Shown are single plots for each marker used in the cluster analysis, displaying the relative antigen expression (x axis) of the DLI cell products for each of the clusters and for all the cells together stratified by response to DLI treatment (A continuous remission, B relapse after DLI).


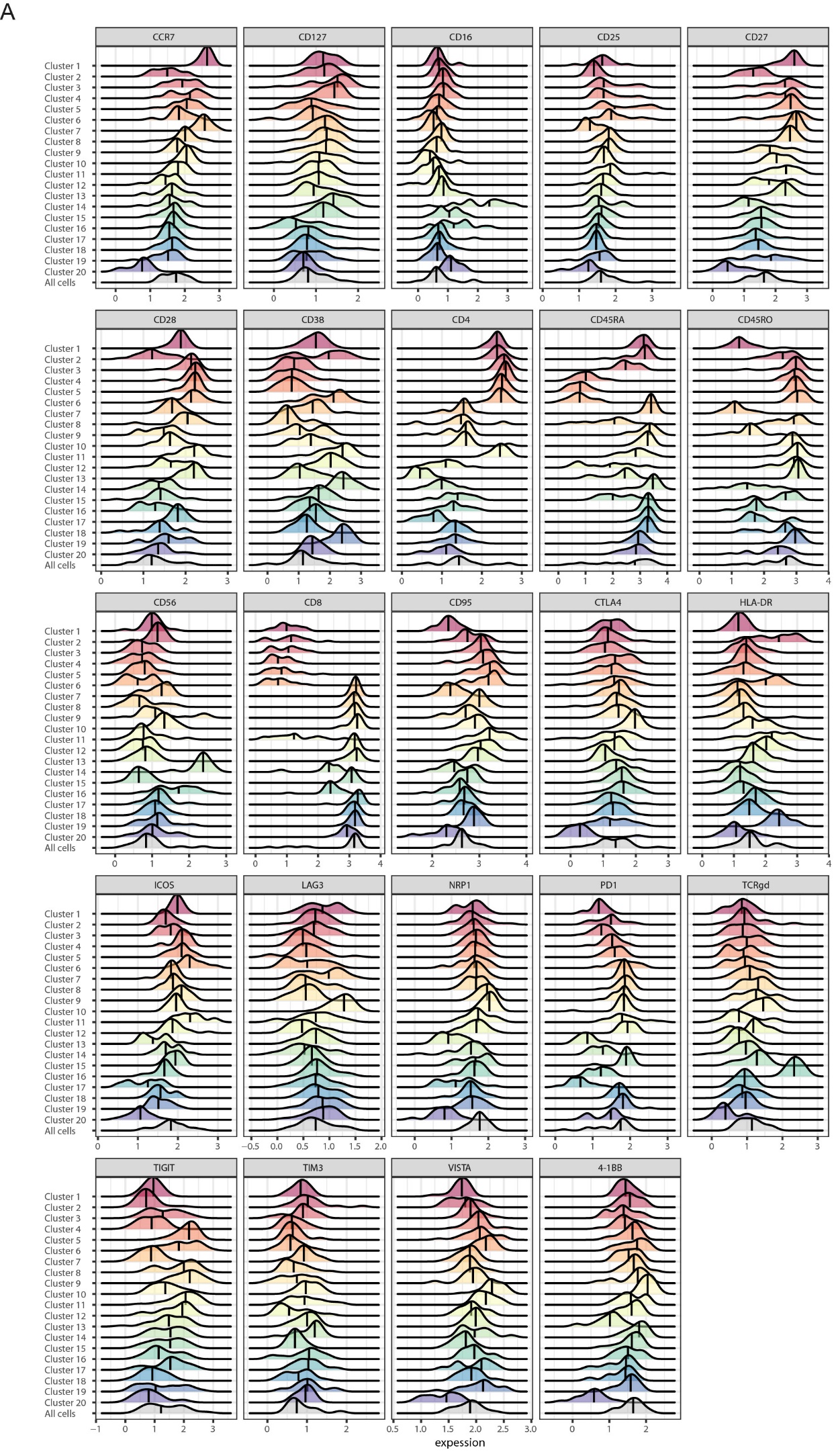


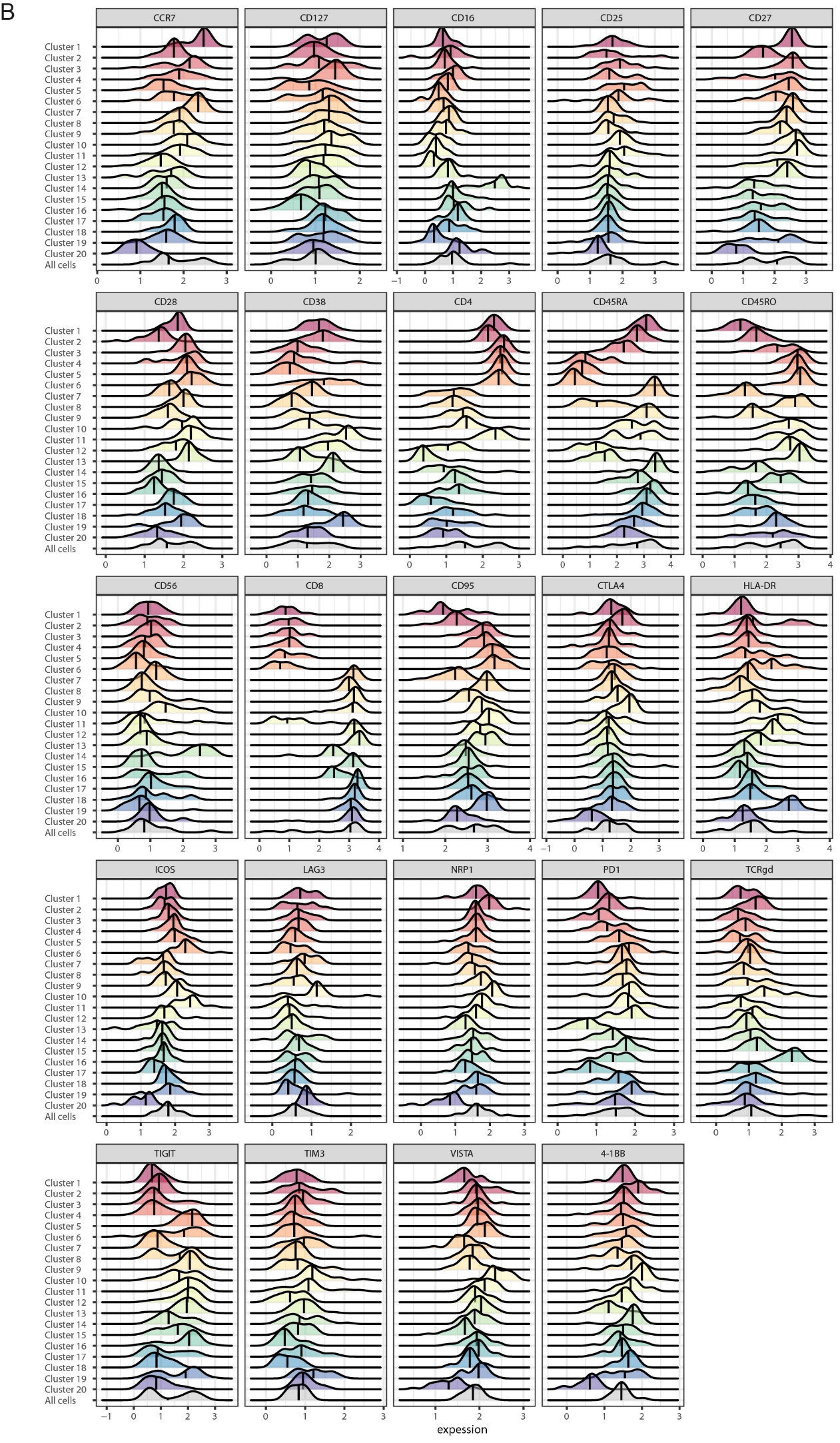
**Supplementary Figure 6. Ridgeplots of patient samples stratified by response to DLI.**

Shown are single plots for each marker used in the cluster analysis, displaying the relative antigen expression (x axis) of the patient samples for each of the clusters and for all the cells together stratified by response to DLI treatment (A continuous remission, B relapse after DLI).

**
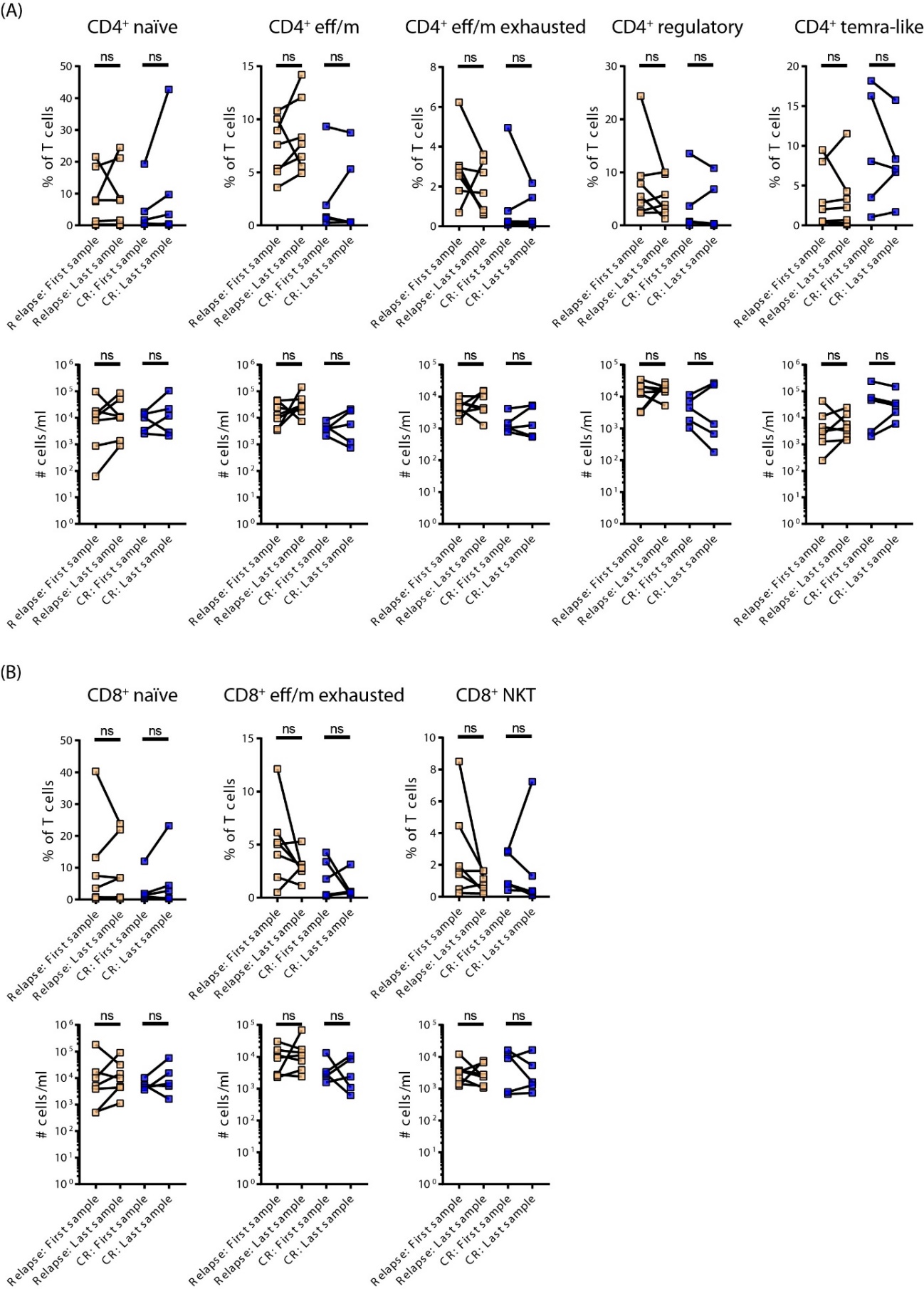
Supplementary Figure 7. Longitudinal comparison of T cell clusters comparing first vs. last sampling time points.**

Longitudinal comparison of T cell clusters, as identified in the UMAP analysis described in Figures 3A and B, comparing first samples post DLI (median time point 25 days (11-84 days) post infusion, Figure 1A and Supplementary Table S7) vs. last samples post DLI (median time point 144 days (27-198 days) post infusion, Figure 1 A and Supplementary Table S8) in patients with continuous remission (CR, blue, n=5) and relapse (orange, n=7) during the 24 months study follow-up. (A) Shows results for five different CD4^+^ T cell clusters and (B) shows results for three different CD8^+^ T cell clusters. Clusters are named as in Figure 3A. Statistical analysis was performed by Student t test or Mann-Whitney test (two-tailed). Abbreviation: CR continuous remission, ns non-significant.

|  | Specificity | Fluorochrome | RRID | Clone | Order number | Company | Dilution |
| --- | --- | --- | --- | --- | --- | --- | --- |
| 1 | CD45RA | BUV395 | AB_2740037 | HI100 | 740298 | BD | 1:100 |
| 2 | CD16 | BUV496 | AB_2870224 | 3G8 | 612944 | BD | 1:100 |
| 3 | Nrp1 | BUV563 | AB_2873486 | U21-1238 | 749094 | BD | 1:20 |
| 4 | CCR5 | BUV615 | AB_2875581 | 3A9 | 751586 | BD | 1:20 |
| 5 | 4-1BB | BUV661 | AB_2871042 | 4B4-1 | 741642 | BD | 1:20 |
| 6 | CD56 | BUV737 | AB_2813880 | NCAM16.2 | 612766 | BD | 1:20 |
| 7 | CD27 | BUV805 | AB_2873108 | L128 | 748704 | BD | 1:20 |
| 8 | LAG3 | BV421 | AB_2629797 | 11C3C65 | 369314 | BioLegend | 1:20 |
| 9 | CD45RO | Pacific Blue | AB_493659 | UCHL1 | 304216 | BioLegend | 1:20 |
| 10 | ICOS | BV480 | AB_2743582 | DX29 | 746248 | BD | 1:20 |
| 11 | CD28 | BV510 | AB_2562030 | CD28.2 | 302936 | BioLegend | 1:20 |
| 12 | HLA-DR | BV570 | AB_2650882 | L243 | 307638 | BioLegend | 1:20 |
| 13 | TCRVg9 | BV605 | AB_2741946 | B3 | 744036 | BD | 1:20 |
| 14 | CD4 | Qdot605 | AB_11180611 | S3.5 | Q10008 | Thermo Fisher | 1:100 |
| 15 | CCR3 | BV711 | AB_2741255 | 5E8 | 743061 | BD | 1:20 |
| 16 | CCR7 | BV785 | AB_2563630 | G043H7 | 353230 | BioLegend | 1:20 |
| 17 | PD1 | FITC | AB_2572163 | NAT105 | 367412 | BioLegend | 1:20 |
| 18 | CD8 | SparkBlue550 | AB_2819983 | SK1 | 344760 | BioLegend | 1:100 |
| 19 | CD3 | AF532 | AB_11218675 | UCHT1 | 58-0038-42 | Thermo Fisher | 1:100 |
| 20 | CD14 | BB700 | AB_2739737 | MφP9 | 566465 | BD | 1:20 |
| 21 | CD38 | PerCP-eFluor710 | AB_1834399 | HB7 | 46-0388-42 | Invitrogen | 1:20 |
| 22 | CD25 | PE | AB_314276 | BC96 | 302606 | BioLegend | 1:20 |
| 23 | TIGIT | PE-eFluor610 | AB_2723715 | MBSA43 | 61-9500-42 | Thermo Fisher | 1:20 |
| 24 | CD95 | PE-Cy5 | AB_314548 | DX2 | 305610 | BioLegend | 1:20 |
| 25 | CTLA4 | PE-Cy7 | AB_2563098 | L3D10 | 349914 | BioLegend | 1:20 |
| 26 | TCR γ/δ | APC | AB_2733463 | 11F2 | 130-113-500 | Miltenyi Biotec | 1:50 |
| 27 | VISTA | AF647 | AB_2744493 | MIH65.rMAb | 566671 | BD | 1:20 |
| 28 | CD127 | AF700 | AB_2566200 | A019D5 | 351344 | BioLegend | 1:20 |
| 29 | Viability | Zombie NIR | n/a | n/a | 423106 | BioLegend | 1:400 |
| 30 | Tim3 | APC-Fire 750 | AB_2632856 | F38-2E2 | 345044 | BioLegend | 1:20 |

**Supplementary Table S1. Antibody panel for spectral flow cytometry.**

**Supplementary Table S2. Disease and transplant characteristics.**

| Patient ID | Patient sex | Age at DLI (years) | Disease risk according to ELN | Disease details | Donor sex | Donor age at alloHSCT (years) | Graft source |
| --- | --- | --- | --- | --- | --- | --- | --- |
| 34 | m | 61 | Adverse | AML | m | 35 | PBSC |
| 36 | f | 24 | Favourable | AML | m | 29 | PBSC |
| 38 | f | 68 | Intermediate | AML | m | 31 | PBSC |
| 41 | f | 22 | Intermediate | AML | f | 27 | PBSC |
| 42 | f | 51 | Favourable | AML | f | 39 | PBSC |
| 45 | m | 57 | Adverse | sAML from MDS | m | 24 | PBSC |
| 47 | m | 60 | Intermediate | AML | m | 58 | PBSC |
| 48 | m | 32 | Favourable | AML | f | 33 | PBSC |
| 49 | f | 59 | Adverse | AML | f | 43 | PBSC |
| 52 | m | 57 | Adverse | sAML from MDS | m | 36 | PBSC |
| 53 | f | 44 | Intermediate | AML | m | 28 | PBSC |
| 54 | f | 75 | Favourable | AML | m | 30 | PBSC |
| 56 | m | 41 | Intermediate | AML | m | 41 | PBSC |

Abbreviations: alloHSCT allogeneic hematopoietic stem cell transplantation; AML acute myeloid leukemia; DLI donor lymphocyte infusion; ELN European Leukemia Net; f female; m male; MDS myelodysplastic syndrome; PBSC peripheral blood stem cells; sAML secondary acute myeloid leukemia.

**Supplementary Table S3. Details about response to DLI.**

| Patient ID | First GVL (months post DLI) | Relapse post DLI (months post DLI) | Antineoplastic treatment post DLI (months post DLI) | Study endpoint: details | Study endpoint/ last follow-up (months post DLI) | Overall GVL response to DLI |
| --- | --- | --- | --- | --- | --- | --- |
| 34 | noGVL | 1 | Hydroxycarbamide (1-2), Aza (1-2) | Death: PD, infection | 2 | Relapse |
| 36 | noGVL | 1 | Hydroxycarbamide (2), Decitabine (2), GO-A-HAM (2-3), TBI- Fludarabine (3) | 2^nd^ alloHSCT | 3 | Relapse |
| 38 | 2 | - | Sora (1-2) | Death: secondary malignancy | 44 | Continuous remission |
| 41 | 3 | - | Aza (1), Sora (1-4) | - | 55 | Continuous remission |
| 42 | noGVL | 2 | Aza (1-4) | Death: PD, infection | 4 | Relapse |
| 45 | 2 | - | - | Death: GVHD, infection | 10 | Continuous remission |
| 47 | 2 | - | - | - | 48 | Continuous remission |
| 48 | noGVL | 1 | Midostaurin (2-5), Aza (3+5+6), FLA-V-IDA (5), Sora (6), FLAMSA-Bu (6-7) | 2^nd^ alloHSCT | 7 | Relapse |
| 49 | noGVL | 6 | Aza (2), Midostaurin (2-3+5),  Sora (9-15) | Death: GVHD, infection | 15 | Relapse |
| 52 | 2 | 20 | Aza (1-2) | Death: PD, infection | 22 | Relapse |
| 53 | 3 | 13 | Aza (1-2+13),  Ven (13+15), Decitabine (15) | - | 39 | Relapse |
| 54 | 3 | - | Aza (1-3), Ven (2-3) | - | 37 | Continuous remission |
| 56 | 1 | 14 | Aza (1-3+14),  Sora (1-12+14-32), irradiation (15) | - | 34 | Relapse |

Abbreviations: alloHSCT allogeneic hematopoietic stem cell transplantation; Aza Azacitidine, Go-A-HAM Gemtuzumab+ Ozogamicin+ Cytarabine+ Mitoxantrone +Tretinoin; GVHD Graft-versus-host-disease; GVL Graft-versus-leukemia; PD progressive disease; R Relapse; Sora Sorafenib, TBI- Fludarabine total body irradiation + Fludarabine; Ven Venetoclax.

**Supplementary Table S4: Cluster phenotypes of DLI cell products.**

| # | Cluster name | Main Phenotype | Reference |
| --- | --- | --- | --- |
| 1 | CD4^+^ reg | CD4^+^ CCR7^+++.^ CD25^++^ CD127^-/lo.^ | (2,3) |
| 2 | CD4^+^ eff/m | CD4^+^ CD45RA^-^ CCR7^-/lo.^ CD27^+^CD28^+^ | (4,5) |
| 3 | CD4^+^ temra | CD4^+^ CD45RA^+^ CCR7^-/+^ CD27^-/+.^ CD28^+^ CD95^+^ | (4,5) |
| 4 | CD4^+^CD8^+^ | CD4^+^ CD8^+^ CD25^++^ CD127^++^ | (6) |
| 5 | CD4^+^ naïve | CD4^+^ CD45RA^+^ CCR7^++^ CD27^++^ | (4–6) |
| 6 | CD4^+^ CD16^+^ CD45RO^+^ | CD4^+^ CD16^+^ CD45RO^++^ CCR7^-^ | (7) |
| 7 | CD8^+^ CD16^+^ CD45RO^+^ | CD8^+^ CD16^+^ CD45RO^+^ CCR7^-^ | (7–9) |
| 8 | CD8^+^ eff/m | CD8^+^ CD45RO^+^ CCR7^-/lo^ CD95^++^ CD27^-/+^ PD1^++/+^ Tim3^-/+^ | (4,10) |
| 9 | CD4^+^ temra HLA-DR^+^ | CD4^+^ CD45RA^+^ CCR7^+^ HLA-DR^+^ CD95^+^ PD1^+^ CD27^-/+^ | (4,11) |
| 10 | CD8^+^ temra | CD8^+^ CD45RA^+^ CCR7^int.^ VISTA^int.^ CD95^int.^ CD27^int.^ | (4,10) |
| 11 | γδ^+^ | γδ^+^ CD4^-^CD8+^-^ | (6) |
| 12 | CD4^+^ CD16^+^ CD27^+^ | CD4^+^ CD45RA^+^ CCR7^++^ CD16^+^ CD56^-/+^ CD95^-^ | (7–9) |
| 13 | CD8^+^ naïve | CD8^+^ CCR7^++^ CD45RA^+^ CD27^++^ CD95^-^ | (4,6,10) |
| 14 | CD8^+^ temra HLA-DR^+^ | CD8^+^ CD45RA^+^ CCR7^-/lo^ CD38^+^ HLA-DR^+^ | (12) |
| 15 | CD8^+^ CD16^+^ CD56^+^ | CD8^+^CD16^+^ CD56^+^ CD45RA^+^ CD27^-^ CCR7^+^ CD95^+^ | (7) |
| 16 | CD8^+^ CD16^+^ CD56^-^ | CD8^+^ CD16^+^ CD56^-^ CD45RA^+^ CD27^+^ CCR7^-/+^ CD95^-^ | (7–9) |
| 17 | CD8^+^ CD16^+^ TIGIT^+^ | CD8^+^ CD16^+^ CD56^-^ CCR7^-^ CD45RA^+^ Tigit^+^ | (7–9) |

**Supplementary Table S5: Correlation of DLI composition with donor age at alloHSCT.**

| Cell population (frequencies) | Spearman r | P value |
| --- | --- | --- |
| γδ^+^ | -0.400 | 0.176 |
| CD4^+^CD8^+^ | 0.121 | 0.696 |
| CD8^+^ CD16^+^ | -0.440 | 0.135 |
| CD8^+^ temra | -0.308 | 0.306 |
| CD8^+^ eff/m | -0.011 | 0.978 |
| CD8^+^ naïve | -0.687 | 0.012 |
| CD4^+^ Tregs | -0.093 | 0.765 |
| CD4^+^ CD16^+^ | -0.319 | 0.286 |
| CD4^+^ temra | 0.115 | 0.710 |
| CD4^+^ eff/m | 0.429 | 0.146 |
| CD4^+^ naïve | 0.110 | 0.723 |

Abbreviations: alloHSCT allogeneic hematopoietic stem cell transplantation.

**Supplementary Table S6. Cluster phenotypes of patients’ samples.**

| # | Cluster name | Main Phenotype | Reference |
| --- | --- | --- | --- |
| 1 | CD4^+^ naïve | CD4^+^ CD45RA^+^ CCR7^++^ CD27^++^ | (4–6) |
| 2 | CD4^+^ temra | CD4^+^ CD45RA^+^ CD45RO^+^ CCR7^+^ CD27^-^ CD28^-^ | (4,5) |
| 3 | CD4^+^ temra CD45RO^+^ | CD4^+^ CD45RO^+^ CCR7^-/lo^ CD27^++^ CD28^++^ | (4) |
| 4 | CD4^+^ eff/m | CD4^+^ CD45RO^+^ CD28^+^ CD27^+^ CD95^+^ | (4,5) |
| 5 | CD4^+^ reg | CD4^+^ CCR7^+++.^ CD25^++^ CD127^-/lo.^ | (2,3) |
| 6 | CD4^+^ eff/m exhausted | CD4^+^ CD45RO^+^ CCR7^-^ PD1^+^HLA-DR^+^ CD38^+^ CD95^++^ | (3,4) |
| 7 | CD8^+^ naïve | CD8^+^ CCR7^++^ CD45RA^+^ CD27^++^ CD95^-^ | (4,6,10) |
| 8 | CD8^+^ eff/m CD27^+^ PD1^+^ | CD8^+^ CD45RO^+^ CD28^+^ CD27^+^ CD95^-/lo^ Tigit^++^ PD1^+^ | (4,6,10) |
| 9 | CD8^+^ temra PD1^+^ | CD8^+^ CD45RA^+^ CCR7^-/lo^ CD28^-^Tigit^+^ CD95^-^ | (4,6,10) |
| 10 | CD8^+^ temra exhausted | CD8^+^ CD45RA^+^ CD45RO^+^ CCR7^-/lo^ CD28^-^Tigit^-^ CD95^+^ LAG3^+^ VISTA^+^ | (4,10,13) |
| 11 | CD4^+^ temra exhausted | CD4^+^ CD45RO^-/+^ CD45RA^-/+^ LAG3^+^ CD95^++^ VISTA^+^ CD38^+^ | (4,13) |
| 12 | CD8^+^ eff/m exhausted | CD8^+^ CD45RA^-^ CD45RO^+^ HLA-DR^+^ CD38^+^ Tigit^+^ PD1^++^ | (4,10,13) |
| 13 | CD8^+^ eff/m PD1^-^ | CD8^+^ CD45RO^+^ CD28^+^ CD27^+^ CD95^-/lo^ Tigit^+^ PD1^-^ | (4,10) |
| 14 | CD8^+^ CD16^+^ CD56^+^ | CD8^+^ CD16^+^ CD56^+^ | (7,14) |
| 15 | CD8^+^ eff/m PD1^+^ | CD8^+^ CD45RA^-/+^ CD45RO^-/+^ PD1^+^ CD38^-/+^ | (4,10,13) |
| 16 | γδ^+^ | γδ^+^ CD4^-^CD8+^-^ | (6) |
| 17 | CD8^+^ temra CD27^-^ | CD8^+^ CD45RA^+^ CCR7^-^CD27^-^ CD95^-^ | (4,10,13) |
| 18 | CD8^+^ temra CD45RO^+^ | CD8^+^ CD45RA^+^ CD45RO^+^ CCR7^-^ | (4,10,13) |
| 19 | CD8^+^ temra CD45RO^+^ HLA-DR^+^ CD38^+^ | CD8^+^ CD45RO^+^ CD45RA^+^  CD38^+^ HLA-DR^+^ PD1^+^ | (4,10,13) |
| 20 | CD8^+^ temra CD27^-^ CD28^-^PD1^-^ | CD8^+^ CD45RA^+^ CD27^-^ CD28^-^ | (4,10,13) |

**Supplementary Table S7: Clinical characteristics of first samples post DLI.**

|  | Relapse  (n=8) | Continuous remission  (n=5) | P- value |
| --- | --- | --- | --- |
| Sampling time point, days post DLI | 28 (12-55) | 18 (11-84) | 0.649 |
| Antineoplastic treatment at sampling time point | 3 (38) | 1 (20) | >0.999 |
| GVHD at sampling time point | 3 (38) | 0 (0) | 0.231 |
| Relapse/GVL at sampling time point | 1 (13) | 0 (0) | n/a |
| Days until first Relapse/GVL at sampling time point | 150 (8-559) | 13 (4-51) | n/a |

Given is the median (range) for continuous variables and the absolute number (%) for categorical variables. Abbreviations: DLI donor lymphocyte infusion; GVHD Graft-versus-host-disease; GVL Graft-versus-leukemia.

**Supplementary Table S8: Clinical characteristics of last samples post DLI.**

|  | Relapse  (n=7) | Continuous remission  (n=5) | P- value |
| --- | --- | --- | --- |
| Sampling time point, days post DLI | 133 (27-198) | 120 (56-170) | 0.500 |
| Antineoplastic treatment at sampling time point | 2 (29) | 1 (20) | >0.999 |
| GVHD at sampling time point | 3 (43) | 3 (60) | >0.999 |
| Relapse/GVL at sampling time point | 4 (57) | 5 (100) | n/a |
| Days until first Relapse/GVL at sampling time point | 211 (186-396) | - | n/a |

Given is the median (range) for continuous variables and the absolute number (%) for categorical variables. Abbreviations: DLI donor lymphocyte infusion; GVHD Graft-versus-host-disease; GVL Graft-versus-leukemia.

**References**

1. Schultze-Florey CR, Kuhlmann L, Raha S, Barros-Martins J, Odak I, Tan L, Xiao Y, Ravens S, Hambach L, Venturini L, et al. Clonal expansion of CD8+ T cells reflects graft-versus-leukemia activity and precedes durable remission following DLI. *Blood Adv* (2021) 5:4485–4499. doi: 10.1182/bloodadvances.2020004073

2. Rodríguez-Perea AL, Arcia ED, Rueda CM, Velilla PA. Phenotypical characterization of regulatory T cells in humans and rodents. *Clin Exp Immunol* (2016) 185:281–91. doi: 10.1111/cei.12804

3. Smigiel KS, Richards E, Srivastava S, Thomas KR, Dudda JC, Klonowski KD, Campbell DJ. CCR7 provides localized access to IL-2 and defines homeostatically distinct regulatory T cell subsets. *J Exp Med* (2014) 211:121–136. doi: 10.1084/jem.20131142

4. Mousset CM, Hobo W, Woestenenk R, Preijers F, Dolstra H, van der Waart AB. Comprehensive Phenotyping of T Cells Using Flow Cytometry. *Cytom Part A* (2019) 95:647–654. doi: 10.1002/cyto.a.23724

5. Sallusto F, Lenig D, Förster R, Lipp M, Lanzavecchia A. Two subsets of memory T lymphocytes with distinct homing potentials and effector functions. *Nature* (1999) 401:708–712. doi: 10.1038/44385

6. Schultze-Florey CR, Chukhno E, Goudeva L, Blasczyk R, Ganser A, Prinz I, Förster R, Koenecke C, Odak I. Distribution of major lymphocyte subsets and memory T-cell subpopulations in healthy adults employing GLP-conforming multicolor flow cytometry. *Leukemia* (2021) 35:3021–3025. doi: 10.1038/s41375-021-01348-5

7. Krijgsman D, Hokland M, Kuppen PJK. The Role of Natural Killer T Cells in Cancer—A Phenotypical and Functional Approach. *Front Immunol* (2018) 9: doi: 10.3389/fimmu.2018.00367

8. Liu L, Chang YJ, Xu LP, Zhang XH, Wang Y, Liu KY, Huang XJ. Reversal of T Cell Exhaustion by the First Donor Lymphocyte Infusion Is Associated with the Persistently Effective Antileukemic Responses in Patients with Relapsed AML after Allo-HSCT. *Biol Blood Marrow Transplant* (2018) 24:1350–1359. doi: 10.1016/j.bbmt.2018.03.030

9. Dutertre C-A, Bonnin-Gélizé E, Pulford K, Bourel D, Fridman W-H, Teillaud J-L. A novel subset of NK cells expressing high levels of inhibitory FcγRIIB modulating antibody-dependent function. *J Leukoc Biol* (2008) 84:1511–1520. doi: 10.1189/jlb.0608343

10. Martin MD, Badovinac VP. Defining Memory CD8 T Cell. *Front Immunol* (2018) 9: doi: 10.3389/fimmu.2018.02692

11. Lee E, Bacchetti P, Milush J, Shao W, Boritz E, Douek D, Fromentin R, Liegler T, Hoh R, Deeks SG, et al. Memory CD4 + T-Cells Expressing HLA-DR Contribute to HIV Persistence During Prolonged Antiretroviral Therapy. *Front Microbiol* (2019) 10:1–19. doi: 10.3389/fmicb.2019.02214

12. Wang Z, Zhu L, Nguyen THO, Wan Y, Sant S, Quiñones-Parra SM, Crawford JC, Eltahla AA, Rizzetto S, Bull RA, et al. Clonally diverse CD38+HLA-DR+CD8+ T cells persist during fatal H7N9 disease. *Nat Commun* (2018) 9: doi: 10.1038/s41467-018-03243-7

13. Wherry EJ, Kurachi M. Molecular and cellular insights into T cell exhaustion. *Nat Rev Immunol* (2015) 15:486–499. doi: 10.1038/nri3862

14. Kim D-H, Chang W-S, Lee Y-S, Lee K-A, Kim Y-K, Kwon BS, Kang C-Y. 4-1BB Engagement Costimulates NKT Cell Activation and Exacerbates NKT Cell Ligand-Induced Airway Hyperresponsiveness and Inflammation. *J Immunol* (2008) 180:2062–2068. doi: 10.4049/jimmunol.180.4.2062
